# Distantly Related Rotaviruses in Common Shrews, Germany, 2004–2014

**DOI:** 10.3201/eid2512.191225

**Published:** 2019-12

**Authors:** Reimar Johne, Simon H. Tausch, Josephine Grützke, Alexander Falkenhagen, Corinna Patzina-Mehling, Martin Beer, Dirk Höper, Rainer G. Ulrich

**Affiliations:** German Federal Institute for Risk Assessment, Berlin, Germany (R. Johne, S.H. Tausch, J. Grützke, A. Falkenhagen, C. Patzina-Mehling);; Friedrich-Loeffler-Institut, Greifswald-Insel Riems, Germany (M. Beer, D. Höper, R.G. Ulrich);; Deutsches Zentrum für Infektionsforschung, partner site Hamburg-Lübeck-Borstel-Insel Riems, Germany (R.G. Ulrich)

**Keywords:** rotavirus, shrews, common shrew, *Sorex araneus*, virus diversity, rotavirus A, rotavirus C, rotavirus H, tentative species, Germany, viruses, diarrheal disease, epidemiology, zoonoses, next-generation sequencing, phylogeny, enteric infections

## Abstract

We screened samples from common shrews (*Sorex araneus*) collected in Germany during 2004–2014 and identified 3 genetically divergent rotaviruses. Virus protein 6 sequence similarities to prototype rotaviruses were low (64.5% rotavirus A, 50.1% rotavirus C [tentative species K], 48.2% rotavirus H [tentative species L]). Shrew-associated rotaviruses might have zoonotic potential.

Rotaviruses are a major cause of diarrhea in young children, causing an estimated 215,000 deaths worldwide every year (*1*). These viruses are nonenveloped and have a genome consisting of 11 segments of double-stranded RNA (*2*); each segment codes for either 1 of the structural proteins, virus protein (VP) 1–7, or 1 or 2 of the nonstructural proteins (NSPs), NSP1–6. Rotaviruses are classified into species A–I or the tentative species J on the basis of the amino acid sequence similarity of the conserved structural protein VP6 and the conserved nucleotide sequence of the genome segment ends (*3*–*5*). For rotavirus A, further classification into genome segment–specific genotypes has been established (*6*). Rotaviruses can infect a wide diversity of animals, and zoonotic transmission of rotaviruses has been reported (*7*).

Shrews are small insectivorous mammals that have been previously identified as reservoirs for other pathogens (e.g., hantaviruses and *Leptospira* spp.) (*8*–*10*). In this investigation, we aimed to determine whether common shrews (*Sorex araneus*, order Eulipotyphla) are also a reservoir for rotaviruses and, if so, assess the genetic variability of the viruses found in this species.

## The Study

**Figure 1:**
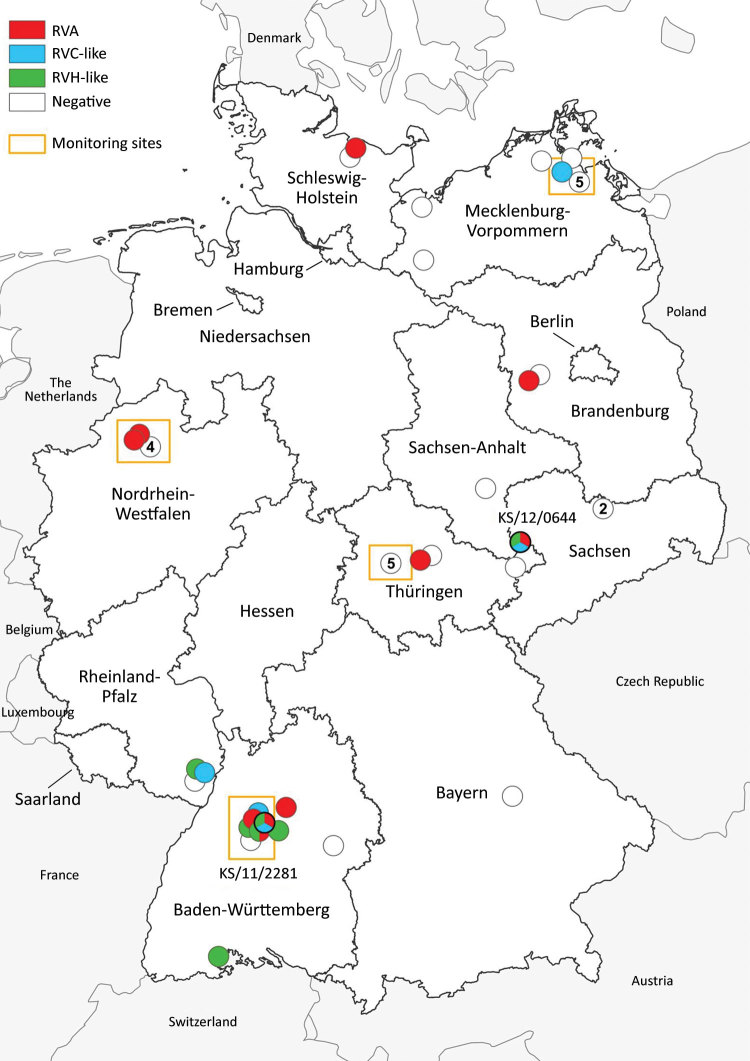
Distribution of common shrews (*Sorex araneus*) collected at monitoring sites (*9*) and additional sites (*10*) in Germany, 2004–2014, positive and negative for RVA, RVC-like, and RVH-like species by reverse transcription PCR. Numbers in white circles indicate the number of negative samples at that collection site; white circles without numbers indicate 1 negative sample at that site. Circles with multiple colors indicate animals with co-infections. The collection sites of the 2 samples analyzed in detail by next-generation sequencing (KS/12/0644 and KS/11/2281; tricolored circles) are indicated. RVA, rotavirus A; RVC, rotavirus C; RVH, rotavirus H.

During 2004–2014, small mammals were caught in different regions of Germany as part of local monitoring or pest control measures (*9*,*10*). From these animal collections, we acquired samples (intestine contents) collected from 49 common shrews (Figure 1). We combined these samples almost equally into 2 pools and performed RNA extraction followed by next-generation sequencing (NGS) using the Ion Torrent Personal Genome Machine system (ThermoFisher Scientific, https://www.thermofisher.com; Appendix). By applying the RIEMS data analysis pipeline (*11*), we identified 3 short contigs with low sequence similarities to rotavirus H in both pools. To identify the positive animals, we extracted RNA from individual samples and screened for rotavirus RNA using reverse transcription PCR (RT-PCR) with primers specific to 1 of the 3 rotavirus H contigs we previously obtained (Appendix Table 1). In total, 7 (15.2%) of 46 samples turned out to be positive for species H–like rotavirus (Table 1); 2 samples, KS/12/0644 and KS/11/2281, generated the strongest signal on ethidium bromide staining. We subjected these 2 samples to RNase and DNase treatment followed by RNA extraction and NGS using the NextSeq 500 sequencing system (Illumina, https://www.illumina.com); 8,576,782 read pairs for KS/12/0644 and 6,168,437 for KS/11/2281 were generated. After a RAMBO-K analysis (*12*) suggested a low abundance of highly deviant rotavirus sequences, we performed data analysis and contig assembly using a newly generated pipeline (Appendix). By this method, contig lengths were 164–3,017 nt, and we obtained 48 contigs with sequence similarities to rotavirus A, 17 with low sequence similarities to rotavirus C, and 23 with low sequence similarities to rotavirus H (Appendix Table 2). Because contigs of homologous genes from each of the 3 viruses were detected in these samples, we concluded 3 different rotaviruses were present in both.

**Table 1:** Rotavirus infections detected in common shrews (*Sorex araneus*) sampled in Germany, 2004–2014*

Virus species	Monoinfections	Co-infections with						Total infections
		RVA	RVC	RVH	RVA and RVC	RVC and RVH	RVA and RVH	
RVA	7/46 (15.2)	NA	0/46	1/46 (2.2)	NA	2/46 (4.3)	NA	10/46 (21.7)
RVC-like	3/46 (6.5)	0/46	NA	0/46	NA	NA	2/46 (4.3)	5/46 (10.9)
RVH-like	4/46 (4.3)	1/46 (2.2)	0/46	NA	2/46 (4.3)	NA	NA	7/46 (15.2)

*Samples from shrews were examined by reverse transcription PCRs specific for RVA, RVC-like, and RVH-like species. Values are no. positive/total (%). NA, not applicable; RVA, rotavirus A; RVC, rotavirus C; RVH, rotavirus H.

We then performed RT-PCR with all samples using primers specific to the species A and species C–like rotavirus contigs from the previous analysis, and 21.7% (10/46) were positive for species A rotavirus and 10.9% (5/46) for species C–like rotavirus; rotavirus co-infections were also identified (Table 1). An analysis of the geographic distribution of shrew rotaviruses in Germany shows that species C–like rotaviruses were mainly located in the northeast and southwest, species H–like rotaviruses mainly in the south, and species A rotaviruses broadly throughout (Figure 1). At the monitoring site in Baden-Württemberg (southwest Germany), frequent detections of different rotaviruses and multiple co-infections were observed.

Despite several efforts, we could delineate only partial genomic sequences of rotaviruses from the NGS data. By application of primer ligation, rapid amplification of cDNA ends, and degenerated primer RT-PCR strategies, we acquired the complete open reading frames of VP1, VP6, and NSP5 of most viruses (Table 2). In addition, we reamplified and sequenced the VP6 genes of all viruses by dideoxy chain–termination sequencing and confirmed the VP6 sequences obtained. Sequence analysis of these genes and in silico translation indicated 14.1%–65.6% amino acid sequence similarity to the respective proteins of other rotavirus species (Table 2). The Rotavirus Classification Working Group reviewed the sequences of the shrew rotavirus A genes in sample KS/11/2281 and designated the new genotypes R23 for VP1, I27 for VP6, and H23 for NSP5. The maximum amino acid sequence similarities to established rotavirus type species of 50.1% for VP6 of species C–like rotavirus and 48.2% (species H) or 48.3% (species J) for VP6 of species H–like rotavirus suggest that these viruses should be classified as novel (tentative) rotavirus species (Table 2).

**Table 2:** Sequence similarities of deduced VP1, VP6, and NSP5 amino acid sequences of rotaviruses from common shrews (*Sorex araneus*), Germany, 2004–2014*

Comparator rotavirus species and strain	Rotavirus species type (shrew sample designation), protein										
	A (KS/11/2281)				C-like (KS/11/2281)				H-like (KS/12/0644)		
	VP1	VP6	NSP5		VP1†	VP6‡	NSP5		VP1	VP6	NSP5
A, SA11	65.6	64.5	47.6		48.6	40.2	24.5		26.8	20.3	17.6
B, WH-1	27.4	17.1	15.9		24.9	14.7	17.6		55.5	39.2	32.9
C, Bristol	48.0	42.8	24.6		63.2	50.1	31.5		25.8	20.0	16.8
D, 05V0059	51.8	39.8	18.2		48.3	36.1	17.8		25.7	20.3	14.5
F, 03V0568	57.5	34.1	20.2		48.5	32.3	22.8		26.0	15.2	17.4
G, 03V0567	26.4	19.1	12.4		25.9	17.1	17.1		56.1	40.7	34.8
H, J19	27.3	17.3	18.8		25.7	17.4	15.1		63.1	48.2	38.2
I, KE135/2012	26.2	17.6	14.1		26.0	13.8	15.9		59.0	44.5	33.3
J, BO4351/Ms/2014	27.0	18.1	15.2		25.2	14.5	14.1		63.0	48.3	43.9

*Values are % sequence similarities. NSP, nonstructural protein; VP, virus protein. †Incomplete at N terminus (≈70 aa residues missing) and C terminus (≈10 aa residues missing). ‡Incomplete at N terminus (≈40 aa residues missing).

**Figure 2:**
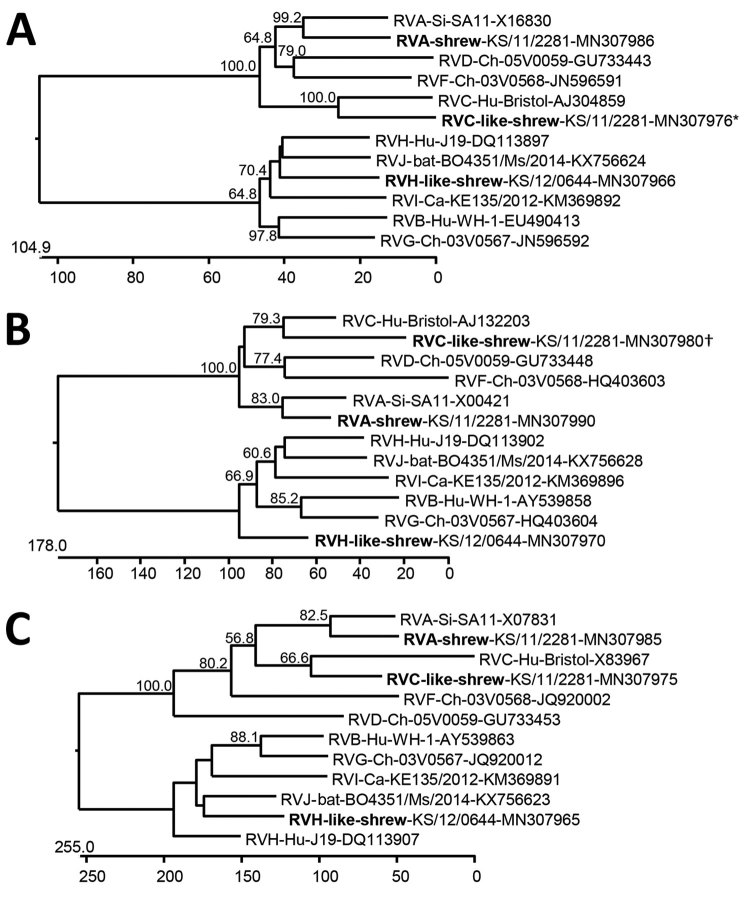
Phylogenetic relationship of shrew rotaviruses (bold), Germany, 2011–2012, with RVA–RVJ determined by using the deduced amino acid sequences of virus protein 1 (A), virus protein 6 (B), and nonstructural protein 5 (C). Trees were constructed by using a neighbor-joining method implemented in the MegAlign module of DNASTAR (https://www.dnastar.com) and a bootstrap analysis with 1,000 trials and 111 random seeds. Bootstrap values of >50% are shown. The rotavirus species, host, strain or sample designation, and GenBank accession number are indicated at each branch. Scale bars indicate amino acid substitutions per 100 residues. *Sequence incomplete at N terminus (≈70 aa residues missing) and C terminus (≈10 aa residues missing). †Sequence incomplete at N terminus (≈40 aa residues missing). Ca, canine; Ch, chicken; Hu, human; RVA, rotavirus A; RVB, rotavirus B; RVC, rotavirus C; RVD, rotavirus D; RVF, rotavirus F; RVG, rotavirus G; RVH, rotavirus H; RVI, rotavirus I; RVJ, rotavirus J; Si, simian.

Phylogenetic analyses of the VP1, VP6, and NSP5 proteins indicate a consistent branching of shrew rotavirus A with other rotavirus A species and shrew species C–like rotavirus with other rotavirus C species. However, the species H–like rotavirus branches more variably within the rotavirus B-G-H-I-J cluster (Figure 2). A more detailed phylogenetic analysis of complete and additional partial genome segment nucleotide sequences of the shrew rotavirus A showed a basal branching at the cluster of other species A rotavirus sequences for most genes (Appendix Figure 1). In addition, phylogenetic analyses of partial amino acid sequences deduced from other genes of the shrew species C–like and H–like rotaviruses confirmed the relationship evident from analyses of the 3 completely sequenced open reading frames (Appendix Figure 2–4).

Shrews have been analyzed infrequently for rotaviruses. In 1 study, rotavirus antigen was detected in wild Chinese tree shrews (*Tupaia chinensis*, order Scandentia) (*13*), and in another study, species A rotaviruses not identical to those of our study (Appendix Figure 1) were identified in house shrews (*Suncus murinus*, order Eulipotyphla) from China (*14*). Here, a broader rotavirus screening of common shrew samples resulted in the identification of novel rotaviruses. The rotavirus detection rate of 10.9%–21.7% in the analyzed samples from animals from different regions of Germany suggests a wide circulation of rotaviruses in shrews, although more samples should be analyzed in the future to clarify the association of rotaviruses with these animals. We also identified co-infections with >1 rotavirus, a regular finding in other animal host species (*15*).

The shrew rotavirus A sequences showed low similarities with other species A rotaviruses, resulting in the assignment of novel genotypes and suggesting a long-term separate evolution of these viruses in this shrew species. The 2 other rotaviruses identified showed even lower sequence similarities to the known rotavirus species. According to the cutoff value of 53% suggested for the differentiation of rotavirus species on the basis of the encoded VP6 amino acid sequence (*5*), both viruses should be considered new rotavirus species, which we tentatively designate rotavirus species K (for the rotavirus C–like species) and L (for the rotavirus H–like species). However, because their complete genome sequences have not been determined, a final classification of these viruses remains to be accomplished. At least the 5′ and 3′ termini of these rotavirus genome segments, which are conserved within known rotavirus species (*2*), should be determined. The low virus amounts in samples, restricted available sample volumes, presence of multiple viruses in single samples, and low sequence similarities for some virus genes might help explain the failure to generate complete genome sequences in our study.

## Conclusions

We identified multiple, genetically divergent rotavirus species in common shrews in Germany. These animals should be further investigated as a potential reservoir for rotaviruses capable of infecting humans.

Appendix. Further information on distantly related rotaviruses in common shrews, Germany, 2004–2014.
