# Supplementary material for: Distantly Related Rotaviruses in Common Shrews, Germany, 2004–2014
Source: Emerg Infect Dis. 2019 Dec;25(12):2310–4. doi: 10.3201/eid2512.191225 (PMC6874240; doi:10.3201/eid2512.191225)
Supplement: Supplementary file 1 — Appendix. Further information on distantly related rotaviruses in common shrews, Germany, 2004–2014. [file 19-1225-Techapp-s1.pdf]

Article DOI: https://doi.org/10.3201/eid2512.191225

# Distantly Related Rotaviruses in Common Shrews, Germany, 2004–2014

## Appendix

## Materials and Methods

### Samples

Within a small mammal monitoring program, shrews were collected as bycatches during 2010–2013 at sites in 4 federal states (*1*). Additional shrews were collected between 2004 and 2014 in different regions of Germany (*2*). Collection of samples was done according to relevant legislation and by permission of the federal authorities (permits Regierungspräsidium Stuttgart 35–9185.82/0261, Landesamt für Natur, Umwelt und Verbraucherschutz Nordrhein-Westfalen 8.87–51.05.20.09.210, Landesamt für Landwirtschaft, Lebensmittelsicherheit und Fischerei Mecklenburg-Vorpommern 7221.3–030/09, Thüringer Landesamt für Lebensmittelsicherheit und Verbraucherschutz 22–2684–04–15–107/09) or were bycatches of the vole monitoring by local forestry institutions as part of their pest control measures. Trapping of the shrews was based on snap trapping, or the shrews were found dead in live traps.

The dissection followed a standard protocol including the collection of intestine samples. For this purpose, the frozen carcasses were thawed, intestinal contents (≈0.2 g) collected, transferred into fresh individual tubes and frozen again at –20°C until further analysis. In this investigation, 49 common shrews (*Sorex araneus*) were included.

### Next-Generation Sequencing (NGS) Analysis of Pooled Samples

Initially, 2 pools were generated: 1 pool of 24 samples was generated by including 6 samples from each of the 4 monitoring sites. A second pool was generated by 25 samples from ongoing monitoring activities in 10 federal states of Germany. RNA was extracted from the pooled samples using the RNeasy Mini kit (QIAGEN, Hilden, Germany). Subsequently, the samples were processed as described (*3*). Briefly, double-stranded cDNA was generated using the cDNA-Synthesis System-Kit (Roche Life Science, Indianapolis, USA) and then transformed

into Ion Torrent compatible libraries. To this end, DNA was fragmented to a peak size of ≈500 bp with a M220 Focused-ultrasonicator (Covaris Inc., Brighton, UK). Then, fragment end-polishing and ligation of adapters (Ion Xpress Barcode Adapters; Life Technologies, Darmstadt, Germany) were performed with a GeneRead DNA Library L Core Kit (QIAGEN). After quality control (Agilent Bioanalyzer 2100 and High Sensitivity DNA Kit; both Agilent, Waldbronn, Germany) and quantification (KAPA Library Quantification Kit - Ion Torrent Personal Genome Machine (PGM) (Uni); Roche, Mannheim, Germany), libraries were pooled and sequenced with an Ion Torrent PGM in 400 bp run using HiQ reagents (Life Technologies, Darmstadt, Germany).

### RT-PCR Screening for Specific Rotaviruses in Fecal Samples

A total of 46 individual samples of intestinal contents (3 of the initial 49 samples had been used up in other experiments) were diluted 1:5 in phosphate-buffered saline (PBS), and 100 µl of the solution was subsequently subjected to nucleic acid extraction using the NucliSENS® easyMAG system (BioMerieux, Marcy I'Etoile, France) according to the manufacturer's instructions. Detection of rotaviruses was done by reverse transcription PCR (RT-PCR) using the One-Step RT-PCR kit (QIAGEN) with primers as described (Appendix Table 1) in a 2720 thermal cycler (Applied Biosystems). The thermal profile comprised 42°C for 30 min and 95°C for 15 min, followed by 40 cycles at 94°C for 30 s, 56°C for 30 s, and 74°C for 40 s, with a final incubation at 74°C for 5 min. PCR products were separated by electrophoresis on ethidium bromide-stained agarose gels.

### NGS Analysis of Samples KS/12/0644 and KS/11/2281

A total of 100 µl of each individual diluted fecal sample was clarified by centrifugation at 9,600 x *g* for 10 min. Thereafter, 70 µl of the supernatant were mixed with 48.5 µl nuclease-free water, 14 µl 10x DNase buffer, 7 µl TurboDNase (Invitrogen), and 0.5 µl RNase A (QIAGEN). After incubation at 37°C for 1 h, RNA was extracted from the sample using the QIAamp viral RNA Mini Kit (QIAGEN) according to the manufacturer's instructions, but without adding carrier-RNA. The RNA was eluted in 60 µl elution buffer (EB) and thereafter subjected to reverse transcription and random amplification using the WTA2 Whole Transcriptome Amplification Kit (Sigma, Deisenhofen, Germany). Briefly, 10 µl of RNA preparation was mixed with 2.5 µl Library Synthesis Solution and 4.1 µl Nuclease-Free Water, heated at 95°C for 2 min, and immediately cooled to 18°C. Thereafter, 2.5 µl Library Synthesis Buffer, 3.9 µl

water, and 2.0 µl Library Synthesis Enzyme were added and incubated at 18°C for 10 min, 25°C for 10 min, 37°C for 30 min, 42°C for 10 min, and 72°C for 20 min, before cooling at 4°C. The following PCR was performed according to the manufacturer's instructions with 10 µl mixture from before in a total volume of 75 µl. The reaction mix was incubated at 94°C for 2 min and 17 cycles at 94°C for 30 sec and 70°C for 5 min each were performed. For NGS, the DNA libraries were generated from the PCR products with the TruSeq® Nano DNA Library Prep Kit (Illumina) and paired-end sequenced with 2 × 151 cycles using the NextSeq 500 sequencing system (Illumina).

The resulting 8,576,782 and 6,168,437 read pairs for samples KS/12/0644 and KS/11/2281 were subjected to trimming with fastp v0.14.1 with default parameters (*4*), yielding 6,498,311 and 4,878,954 read pairs, respectively. For a fast test for the presence of rotavirus reads with low similarity to known references, we classified rotavirus reads using RAMBO-K v1.21 (*5*). As a SPAdes v3.12.0 assembly (*6*) of the selected reads yielded only incomplete rotavirus genomes, we then assembled all trimmed reads using metaSPAdes (*6*). Resulting contigs were searched in the complete NCBI nr database using BLASTX v.2.6.0 with wordsize 4 (*7*). Contigs with hits to any rotavirus sequence were selected as putative rotavirus sequences and subjected to further manual analysis (see below).

**PCR, FLAC, and RACE for Completion of Segment Sequences**

First trials to complete the 5′- and 3′-ends of the genome segments were done by full-length amplification of cDNA (FLAC) as described (*8*). Further trials were done by rapid amplification of cDNA ends (RACE) using the 5′ RACE System Kit (Invitrogen GmbH, Karlsruhe, Germany) as described (*9*). In both cases, specific primers delineated from NGS-derived sequences  were used. In an additional set of experiments, specific RT-PCRs with these primers and those derived from an alignment of segment ends of other available rotavirus sequences were performed to complete the open reading frames for virus protein (VP) 1, VP6, and nonstructural protein 5 (NSP5). The RT-PCR was performed as described above for detection of rotaviruses; however, the cycling conditions were adapted to primer sequences and product lengths. Products with the expected lengths were purified using the QiaQuick DNA Purification Kit (QIAGEN) and subjected to dideoxy chain termination sequencing by a commercial supplier (Eurofins, Ebersberg, Germany).

## Sequence Data Analysis

The NGS contigs were manually inspected using the SeqBuilder module of the DNASTAR software package (Lasergene, Madison, WI, USA) and flanking primer sequences were removed. Sequences were assembled from these NGS contigs and sequences from PCR, FLAC, or RACE products using the same software. The sequences were submitted to the GenBank database and accession numbers MN307962–MN207992 were assigned (also indicated on the branches of the phylogenetic trees). Sequences of the rotavirus type species and the rotavirus A genotype reference strains were used for alignments and phylogenetic comparisons. Nucleotide sequences were used for genotyping and assessment of phylogenetic relationship to other rotavirus A strains in the case of shrew rotavirus A sequences. As only very low nucleotide sequence similarities were evident for the species C-like and H-like rotaviruses of shrews compared with the reference strains, the deduced amino sequences were used, which showed a higher degree of sequence conservation. The phylogenetic trees were constructed using a neighbor-joining method implemented in the MEGALIGN module of the DNASTAR software package (Lasergene), and bootstrap analysis with 1000 trials and 111 random seeds was performed. Genotyping of rotavirus A genome segments was done as described (*10*). According to this protocol, partial sequences can only be assigned to an existing genotype if they have a minimum length of 500 nt and cover more than 50% of the open reading frame. In case they are only distantly related to the established genotypes, the complete sequence of the open reading frame has to be submitted to the Rotavirus Classification Working Group for genotype assignment, which was done for the VP1-, VP6- and NSP5-encoding genome segments of the shrew rotavirus A.

## Appendix References


1. Fischer S, Mayer-Scholl A, Imholt C, Spierling NG, Heuser E, Schmidt S, et al. *Leptospira* genomospecies and sequence type prevalence in small mammal populations in Germany. Vector Borne Zoonotic Dis. 2018;18:188–99. PubMed https://doi.org/10.1089/vbz.2017.2140

2. Mayer-Scholl A, Hammerl JA, Schmidt S, Ulrich RG, Pfeffer M, Woll D, et al. *Leptospira* spp. in rodents and shrews in Germany. Int J Environ Res Public Health. 2014;11:7562–74. PubMed https://doi.org/10.3390/ijerph110807562

3. Wylezich C, Papa A, Beer M, Höper D. A versatile sample processing workflow for metagenomic pathogen detection. Sci Rep. 2018;8:13108. PubMed https://doi.org/10.1038/s41598-018-31496-1

4. Chen S, Zhou Y, Chen Y, Gu J. fastp: an ultra-fast all-in-one FASTQ preprocessor. Bioinformatics. 2018;34:i884–90. PubMed https://doi.org/10.1093/bioinformatics/bty560

5. Tausch SH, Renard BY, Nitsche A, Dabrowski PW. RAMBO-K: rapid and sensitive removal of background sequences from next generation sequencing data. PLoS One. 2015;10:e0137896. PubMed https://doi.org/10.1371/journal.pone.0137896

6. Nurk S, Meleshko D, Korobeynikov A, Pevzner PA. metaSPAdes: a new versatile metagenomic assembler. Genome Res. 2017;27:824–34. PubMed https://doi.org/10.1101/gr.213959.116

7. Morgulis A, Coulouris G, Raytselis Y, Madden TL, Agarwala R, Schäffer AA. Database indexing for production MegaBLAST searches. Bioinformatics. 2008;24:1757–64. PubMed https://doi.org/10.1093/bioinformatics/btn322

8. Kindler E, Trojnar E, Heckel G, Otto PH, Johne R. Analysis of rotavirus species diversity and evolution including the newly determined full-length genome sequences of rotavirus F and G. Infect Genet Evol. 2013;14:58–67. PubMed https://doi.org/10.1016/j.meegid.2012.11.015

9. Schielke A, Sachs K, Lierz M, Appel B, Jansen A, Johne R. Detection of hepatitis E virus in wild boars of rural and urban regions in Germany and whole genome characterization of an endemic strain. Virol J. 2009;6:58. PubMed https://doi.org/10.1186/1743-422X-6-58

10. Matthijnssens J, Ciarlet M, Rahman M, Attoui H, Bányai K, Estes MK, et al. Recommendations for the classification of group A rotaviruses using all 11 genomic RNA segments. Arch Virol. 2008;153:1621–9. PubMed https://doi.org/10.1007/s00705-008-0155-1

**Appendix Table 1.** Primers used for detection of RVA, RVC-like and RVH-like viruses in shrews*

| Virus | Gene | Designation | Sequence (5′-3′) | Product length, bp |
|---|---|---|---|---|
| RVA | VP1 | S-RVA-s | CGTTTCACGTAGGATTCAGGAA | 225 |
| | | S-RVA-as | CTCCATTTGACGCTGATGACAT | |
| RVC-like | VP1 | S-RVC-like-s | AACTGTGTCAAATGACGTCACA | 218 |
| | | S-RVC-like-as | GAACTGTTTCGATCGCACGATT | |
| RVH-like | VP3 | RVH-like-s | CCAGACAACTTACTCATTCTGTTG | 207 |
| | | RVH-like-as | TTGTGTGTGGTTCTCCTTTACCT | |

*RVA, rotavirus A; RVC, rotavirus C; RVH, rotavirus H; VP, virus protein; bp, base pairs.

**Appendix Table 2.** Contig and read numbers and lengths generated by NGS of samples KS/12/0644 and KS/11/2281*

| | KS/12/0644 | | | KS/11/2281 | | |
|---|---|---|---|---|---|---|
| Category | RVA | RVC-like | RVH-like | RVA | RVC-like | RVH-like |
| Contig number | 23 | 7 | 18 | 25 | 10 | 5 |
| Contig lengths | 200–1135 | 193–667 | 374–2584 | 164–821 | 636–3017 | 264–506 |
| Read coverage | 1.6–11.9 | 1.1–9.3 | 3.8–22.4 | 1.1–3.8 | 4.6–19.6 | 1.3–1.9 |
| Total rotavirus read number | | 3597 | | | 2389 | |
| Total read number† | | 8,576,782 | | | 6,168,437 | |

*NGS, next-generation sequencing; RVA, rotavirus A; RVC, rotavirus C; RVH, rotavirus H.
†number of all generated reads including rotavirus reads and non-rotavirus reads from the sample.

A

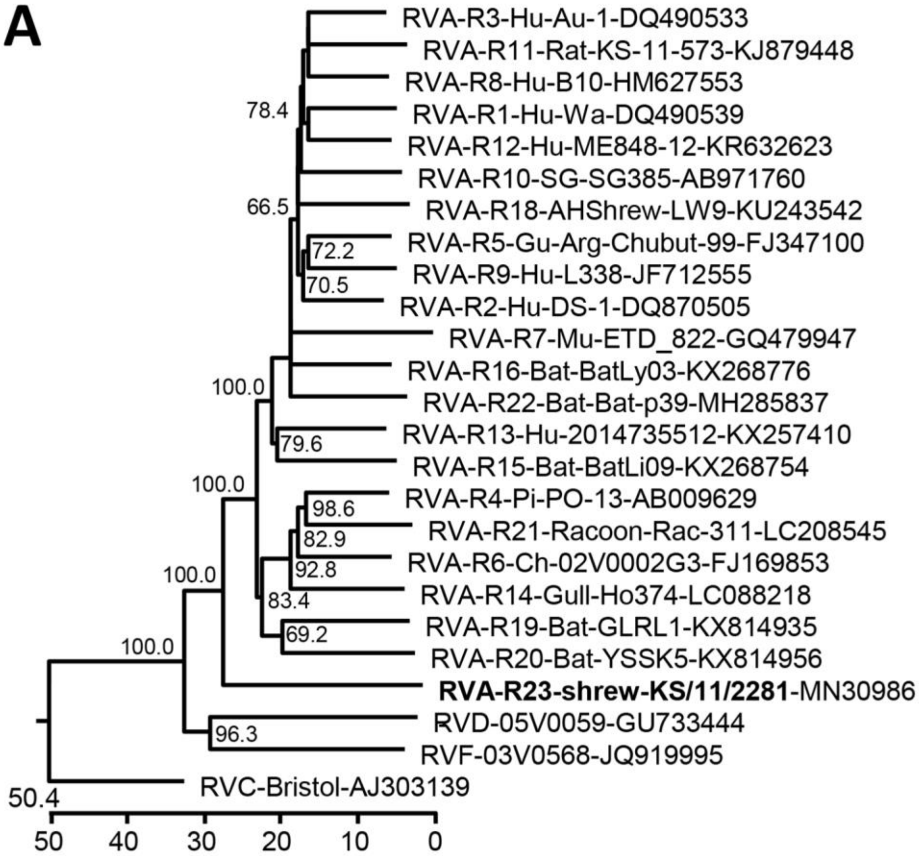
RVA-R3-Hu-Au-1-DQ490533
RVA-R11-Rat-KS-11-573-KJ879448
RVA-R8-Hu-B10-HM627553
78.4
RVA-R1-Hu-Wa-DQ490539
RVA-R12-Hu-ME848-12-KR632623
RVA-R10-SG-SG385-AB971760
66.5
RVA-R18-AHShrew-LW9-KU243542
72.2
RVA-R5-Gu-Arg-Chubut-99-FJ347100
RVA-R9-Hu-L338-JF712555
70.5
RVA-R2-Hu-DS-1-DQ870505
RVA-R7-Mu-ETD_822-GQ479947
RVA-R16-Bat-BatLy03-KX268776
100.0
RVA-R22-Bat-Bat-p39-MH285837
79.6
RVA-R13-Hu-2014735512-KX257410
RVA-R15-Bat-BatLi09-KX268754
100.0
RVA-R4-Pi-PO-13-AB009629
98.6
RVA-R21-Racoon-Rac-311-LC208545
82.9
RVA-R6-Ch-02V0002G3-FJ169853
100.0
92.8
RVA-R14-Gull-Ho374-LC088218
83.4
RVA-R19-Bat-GLRL1-KX814935
69.2
RVA-R20-Bat-YSSK5-KX814956
100.0
**RVA-R23-shrew-KS/11/2281**-MN30986
RVD-05V0059-GU733444
96.3
RVF-03V0568-JQ919995
RVC-Bristol-AJ303139
50.4
50
40
30
20
10
0

B

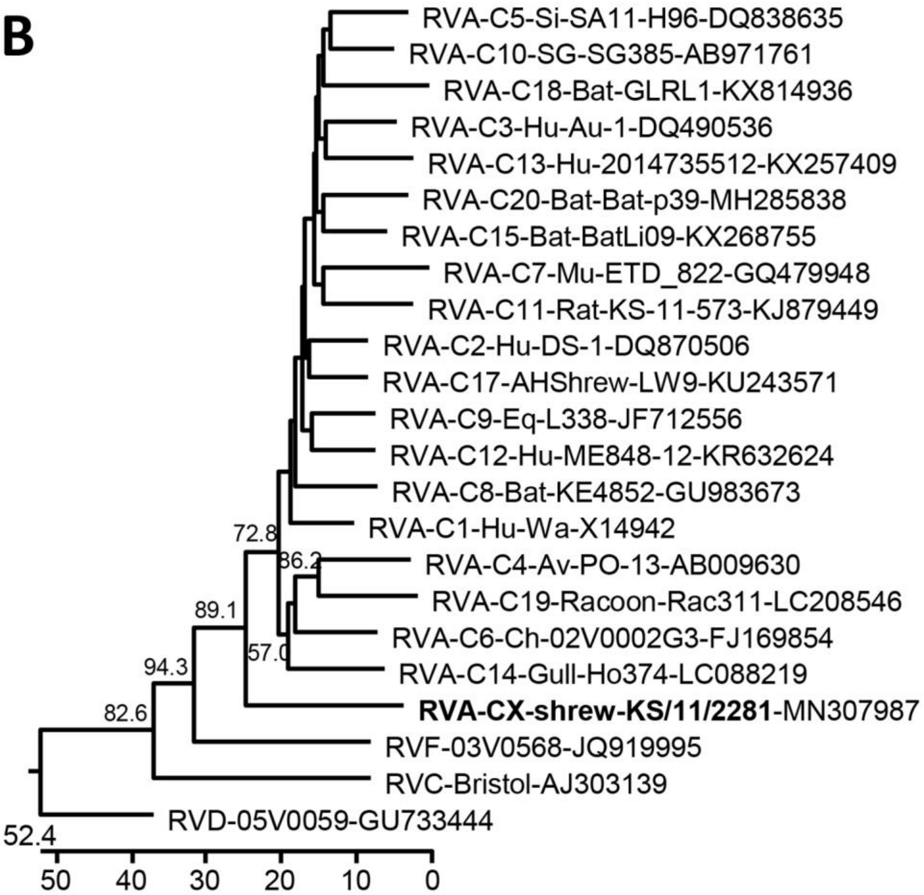
RVA-C5-Si-SA11-H96-DQ838635
RVA-C10-SG-SG385-AB971761
RVA-C18-Bat-GLRL1-KX814936
RVA-C3-Hu-Au-1-DQ490536
RVA-C13-Hu-2014735512-KX257409
RVA-C20-Bat-Bat-p39-MH285838
RVA-C15-Bat-BatLi09-KX268755
RVA-C7-Mu-ETD_822-GQ479948
RVA-C11-Rat-KS-11-573-KJ879449
RVA-C2-Hu-DS-1-DQ870506
RVA-C17-AHShrew-LW9-KU243571
RVA-C9-Eq-L338-JF712556
RVA-C12-Hu-ME848-12-KR632624
RVA-C8-Bat-KE4852-GU983673
RVA-C1-Hu-Wa-X14942
72.8
86.2
RVA-C4-Av-PO-13-AB009630
RVA-C19-Racoon-Rac311-LC208546
89.1
RVA-C6-Ch-02V0002G3-FJ169854
57.0
94.3
RVA-C14-Gull-Ho374-LC088219
82.6
RVA-CX-shrew-KS/11/2281-MN307987
RVF-03V0568-JQ919995
RVC-Bristol-AJ303139
RVD-05V0059-GU733444
52.4
50
40
30
20
10
0

C

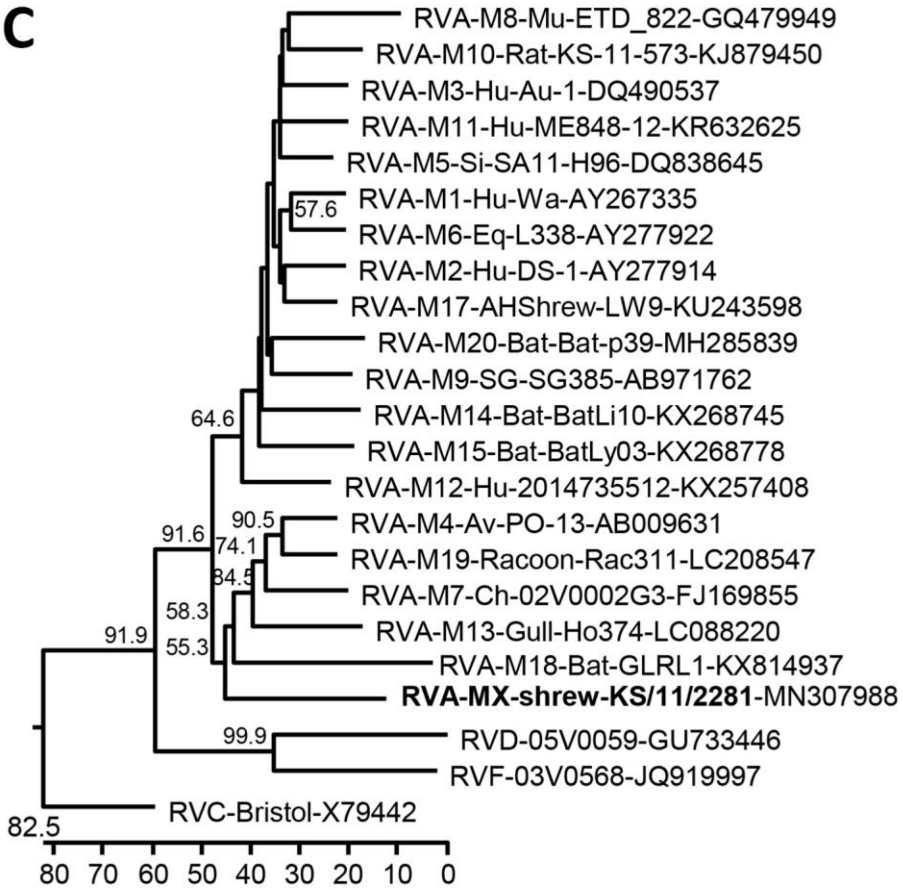

RVA-M8-Mu-ETD_822-GQ479949
RVA-M10-Rat-KS-11-573-KJ879450
RVA-M3-Hu-Au-1-DQ490537
RVA-M11-Hu-ME848-12-KR632625
RVA-M5-Si-SA11-H96-DQ838645
57.6
RVA-M1-Hu-Wa-AY267335
RVA-M6-Eq-L338-AY277922
RVA-M2-Hu-DS-1-AY277914
RVA-M17-AHShrew-LW9-KU243598
RVA-M20-Bat-Bat-p39-MH285839
RVA-M9-SG-SG385-AB971762
64.6
RVA-M14-Bat-BatLi10-KX268745
RVA-M15-Bat-BatLy03-KX268778
RVA-M12-Hu-2014735512-KX257408
90.5
RVA-M4-Av-PO-13-AB009631
91.6
74.1
RVA-M19-Racoon-Rac311-LC208547
84.5
RVA-M7-Ch-02V0002G3-FJ169855
58.3
RVA-M13-Gull-Ho374-LC088220
91.9
55.3
RVA-M18-Bat-GLRL1-KX814937
**RVA-MX-shrew-KS/11/2281**-MN307988
99.9
RVD-05V0059-GU733446
RVF-03V0568-JQ919997
RVC-Bristol-X79442
82.5
80 70 60 50 40 30 20 10 0

D

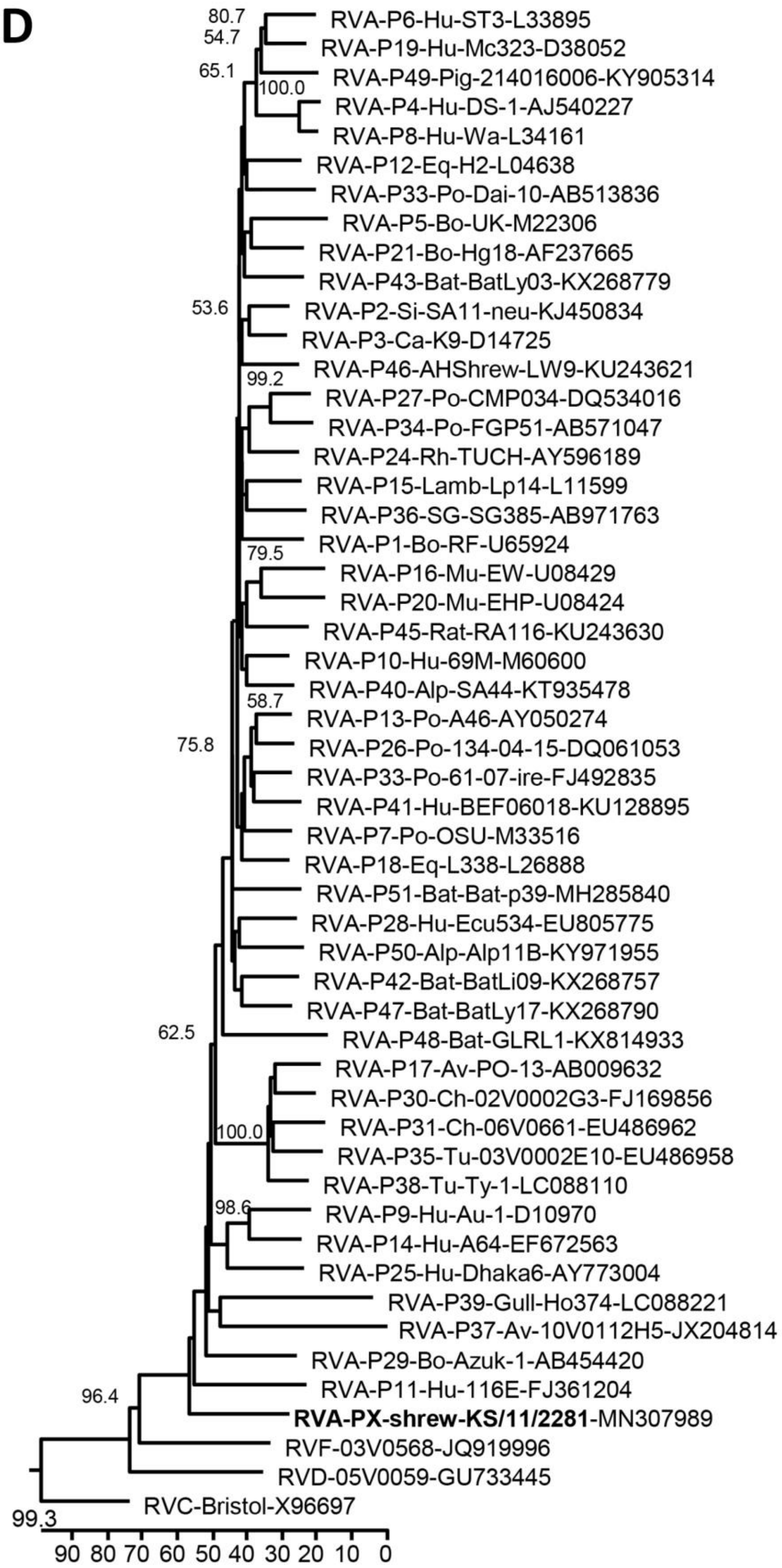

80.7
54.7
65.1
100.0
RVA-P6-Hu-ST3-L33895
RVA-P19-Hu-Mc323-D38052
RVA-P49-Pig-214016006-KY905314
RVA-P4-Hu-DS-1-AJ540227
RVA-P8-Hu-Wa-L34161
RVA-P12-Eq-H2-L04638
RVA-P33-Po-Dai-10-AB513836
RVA-P5-Bo-UK-M22306
RVA-P21-Bo-Hg18-AF237665
RVA-P43-Bat-BatLy03-KX268779
53.6
RVA-P2-Si-SA11-neu-KJ450834
RVA-P3-Ca-K9-D14725
RVA-P46-AHShrew-LW9-KU243621
99.2
RVA-P27-Po-CMP034-DQ534016
RVA-P34-Po-FGP51-AB571047
RVA-P24-Rh-TUCH-AY596189
RVA-P15-Lamb-Lp14-L11599
RVA-P36-SG-SG385-AB971763
RVA-P1-Bo-RF-U65924
79.5
RVA-P16-Mu-EW-U08429
RVA-P20-Mu-EHP-U08424
RVA-P45-Rat-RA116-KU243630
RVA-P10-Hu-69M-M60600
RVA-P40-Alp-SA44-KT935478
58.7
RVA-P13-Po-A46-AY050274
75.8
RVA-P26-Po-134-04-15-DQ061053
RVA-P33-Po-61-07-ire-FJ492835
RVA-P41-Hu-BEF06018-KU128895
RVA-P7-Po-OSU-M33516
RVA-P18-Eq-L338-L26888
RVA-P51-Bat-Bat-p39-MH285840
RVA-P28-Hu-Ecu534-EU805775
RVA-P50-Alp-Alp11B-KY971955
RVA-P42-Bat-BatLi09-KX268757
RVA-P47-Bat-BatLy17-KX268790
62.5
RVA-P48-Bat-GLRL1-KX814933
RVA-P17-Av-PO-13-AB009632
RVA-P30-Ch-02V0002G3-FJ169856
RVA-P31-Ch-06V0661-EU486962
100.0
RVA-P35-Tu-03V0002E10-EU486958
RVA-P38-Tu-Ty-1-LC088110
98.6
RVA-P9-Hu-Au-1-D10970
RVA-P14-Hu-A64-EF672563
RVA-P25-Hu-Dhaka6-AY773004
RVA-P39-Gull-Ho374-LC088221
RVA-P37-Av-10V0112H5-JX204814
RVA-P29-Bo-Azuk-1-AB454420
96.4
RVA-P11-Hu-116E-FJ361204
RVA-PX-shrew-KS/11/2281-MN307989
RVF-03V0568-JQ919996
RVD-05V0059-GU733445
RVC-Bristol-X96697
99.3
90 80 70 60 50 40 30 20 10 0

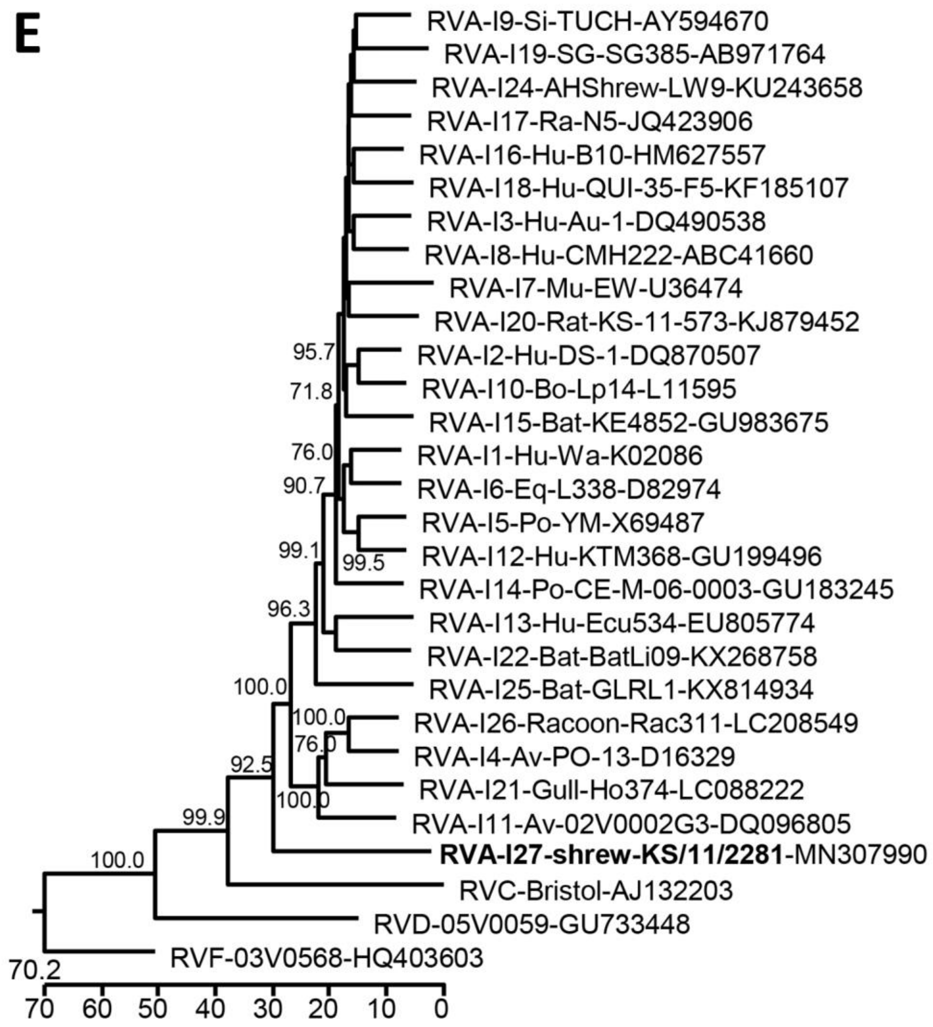
E
RVA-I9-Si-TUCH-AY594670
RVA-I19-SG-SG385-AB971764
RVA-I24-AHShrew-LW9-KU243658
RVA-I17-Ra-N5-JQ423906
RVA-I16-Hu-B10-HM627557
RVA-I18-Hu-QUI-35-F5-KF185107
RVA-I3-Hu-Au-1-DQ490538
RVA-I8-Hu-CMH222-ABC41660
RVA-I7-Mu-EW-U36474
RVA-I20-Rat-KS-11-573-KJ879452
95.7
RVA-I2-Hu-DS-1-DQ870507
71.8
RVA-I10-Bo-Lp14-L11595
RVA-I15-Bat-KE4852-GU983675
76.0
RVA-I1-Hu-Wa-K02086
90.7
RVA-I6-Eq-L338-D82974
RVA-I5-Po-YM-X69487
99.1
99.5
RVA-I12-Hu-KTM368-GU199496
RVA-I14-Po-CE-M-06-0003-GU183245
96.3
RVA-I13-Hu-Ecu534-EU805774
RVA-I22-Bat-BatLi09-KX268758
100.0
RVA-I25-Bat-GLRL1-KX814934
100.0
RVA-I26-Racoon-Rac311-LC208549
76.0
RVA-I4-Av-PO-13-D16329
92.5
RVA-I21-Gull-Ho374-LC088222
100.0
99.9
RVA-I11-Av-02V0002G3-DQ096805
RVA-I27-shrew-KS/11/2281-MN307990
100.0
RVC-Bristol-AJ132203
RVD-05V0059-GU733448
RVF-03V0568-HQ403603
70.2
70 60 50 40 30 20 10 0

**F**

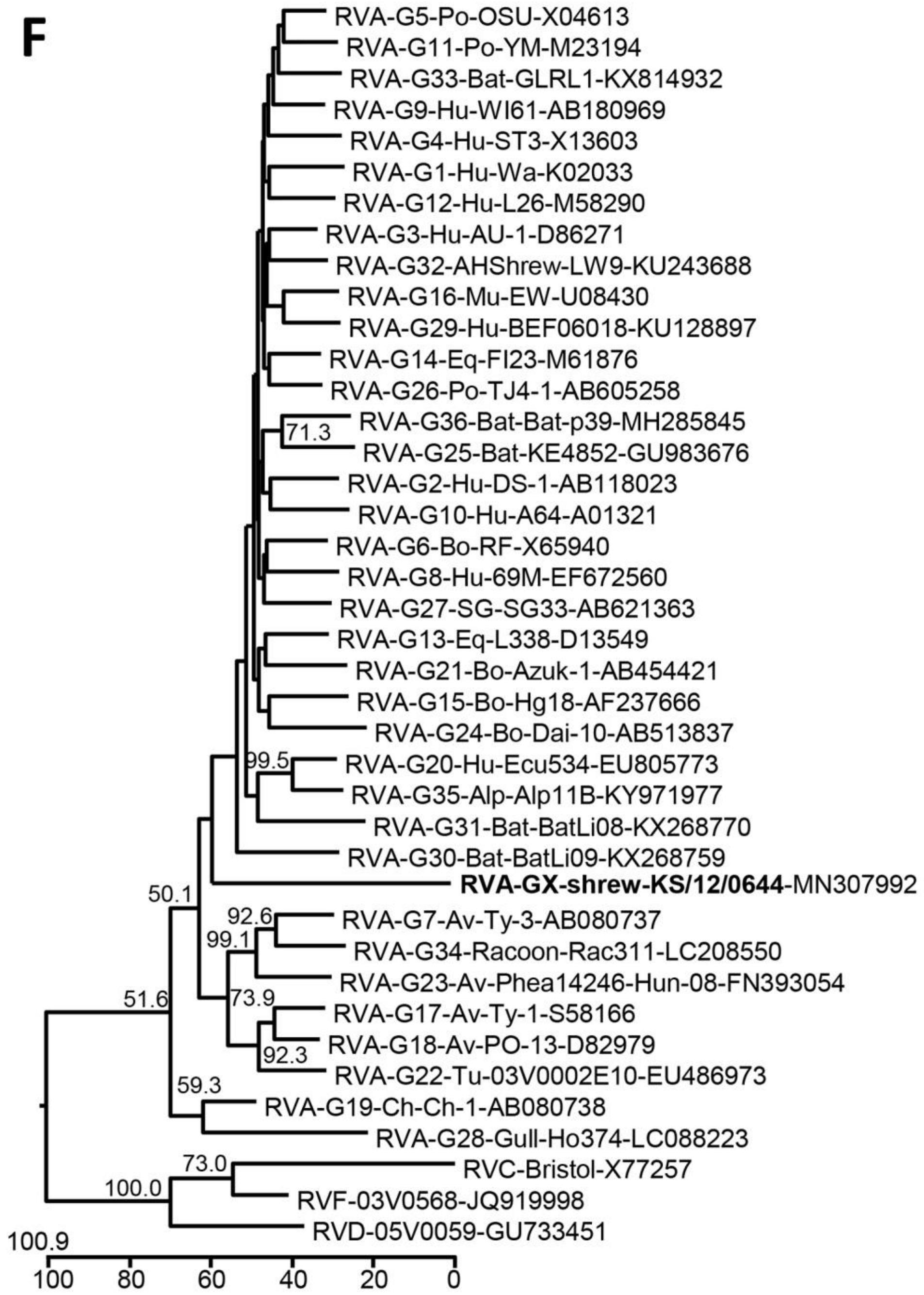

RVA-G5-Po-OSU-X04613
RVA-G11-Po-YM-M23194
RVA-G33-Bat-GLRL1-KX814932
RVA-G9-Hu-WI61-AB180969
RVA-G4-Hu-ST3-X13603
RVA-G1-Hu-Wa-K02033
RVA-G12-Hu-L26-M58290
RVA-G3-Hu-AU-1-D86271
RVA-G32-AHShrew-LW9-KU243688
RVA-G16-Mu-EW-U08430
RVA-G29-Hu-BEF06018-KU128897
RVA-G14-Eq-FI23-M61876
RVA-G26-Po-TJ4-1-AB605258
71.3
RVA-G36-Bat-Bat-p39-MH285845
RVA-G25-Bat-KE4852-GU983676
RVA-G2-Hu-DS-1-AB118023
RVA-G10-Hu-A64-A01321
RVA-G6-Bo-RF-X65940
RVA-G8-Hu-69M-EF672560
RVA-G27-SG-SG33-AB621363
RVA-G13-Eq-L338-D13549
RVA-G21-Bo-Azuk-1-AB454421
RVA-G15-Bo-Hg18-AF237666
RVA-G24-Bo-Dai-10-AB513837
99.5
RVA-G20-Hu-Ecu534-EU805773
RVA-G35-Alp-Alp11B-KY971977
RVA-G31-Bat-BatLi08-KX268770
RVA-G30-Bat-BatLi09-KX268759
RVA-GX-shrew-KS/12/0644-MN307992
50.1
92.6
RVA-G7-Av-Ty-3-AB080737
99.1
RVA-G34-Racoon-Rac311-LC208550
RVA-G23-Av-Phea14246-Hun-08-FN393054
51.6
73.9
RVA-G17-Av-Ty-1-S58166
RVA-G18-Av-PO-13-D82979
92.3
RVA-G22-Tu-03V0002E10-EU486973
59.3
RVA-G19-Ch-Ch-1-AB080738
RVA-G28-Gull-Ho374-LC088223
73.0
RVC-Bristol-X77257
100.0
RVF-03V0568-JQ919998
RVD-05V0059-GU733451
100.9
100
80
60
40
20
0

G

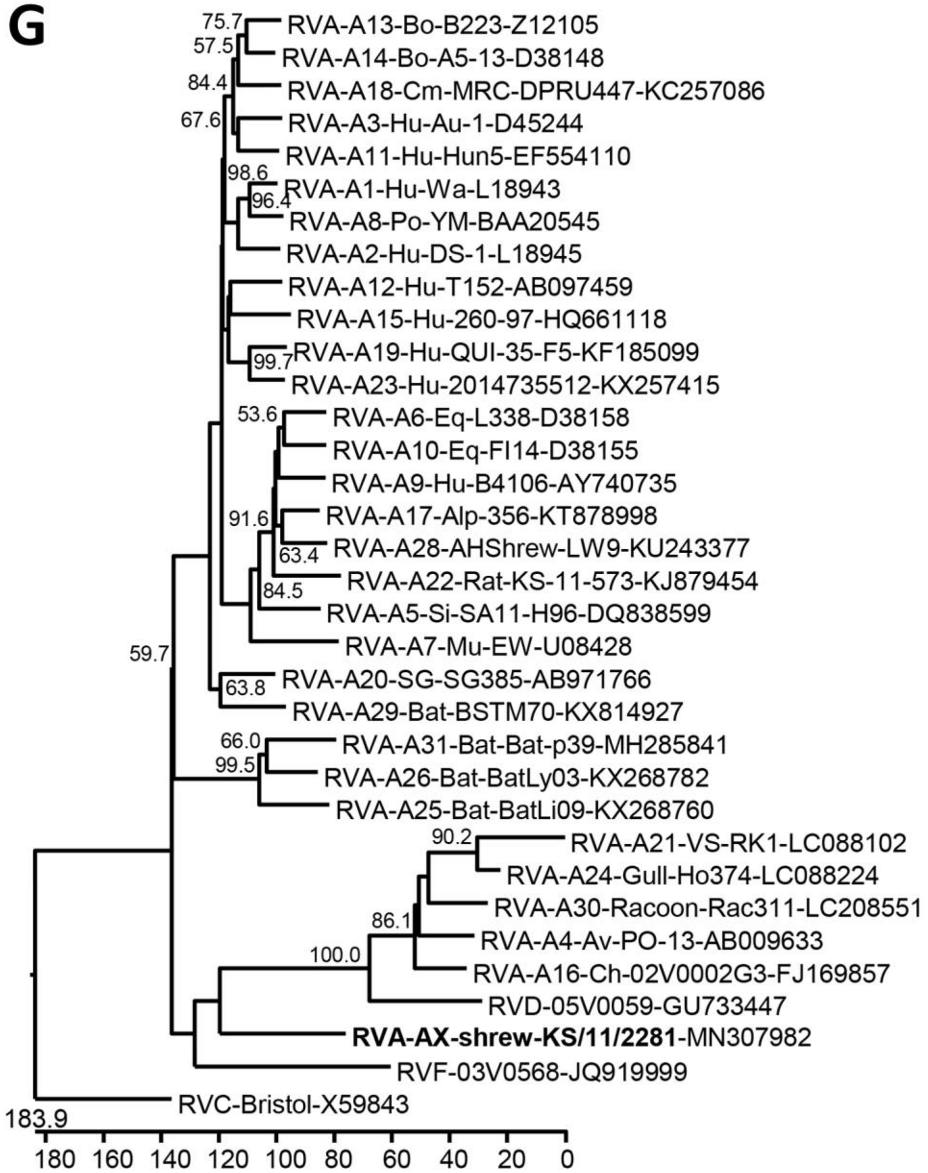
RVA-A13-Bo-B223-Z12105
RVA-A14-Bo-A5-13-D38148
RVA-A18-Cm-MRC-DPRU447-KC257086
RVA-A3-Hu-Au-1-D45244
RVA-A11-Hu-Hun5-EF554110
RVA-A1-Hu-Wa-L18943
RVA-A8-Po-YM-BAA20545
RVA-A2-Hu-DS-1-L18945
RVA-A12-Hu-T152-AB097459
RVA-A15-Hu-260-97-HQ661118
RVA-A19-Hu-QUI-35-F5-KF185099
RVA-A23-Hu-2014735512-KX257415
RVA-A6-Eq-L338-D38158
RVA-A10-Eq-FI14-D38155
RVA-A9-Hu-B4106-AY740735
RVA-A17-Alp-356-KT878998
RVA-A28-AHShrew-LW9-KU243377
RVA-A22-Rat-KS-11-573-KJ879454
RVA-A5-Si-SA11-H96-DQ838599
RVA-A7-Mu-EW-U08428
RVA-A20-SG-SG385-AB971766
RVA-A29-Bat-BSTM70-KX814927
RVA-A31-Bat-Bat-p39-MH285841
RVA-A26-Bat-BatLy03-KX268782
RVA-A25-Bat-BatLi09-KX268760
RVA-A21-VS-RK1-LC088102
RVA-A24-Gull-Ho374-LC088224
RVA-A30-Racoon-Rac311-LC208551
RVA-A4-Av-PO-13-AB009633
RVA-A16-Ch-02V0002G3-FJ169857
RVD-05V0059-GU733447
RVA-AX-shrew-KS/11/2281-MN307982
RVF-03V0568-JQ919999
RVC-Bristol-X59843
75.7
57.5
84.4
67.6
98.6
96.4
99.7
53.6
91.6
63.4
84.5
59.7
63.8
66.0
99.5
90.2
86.1
100.0
183.9
180 160 140 120 100 80 60 40 20 0

H

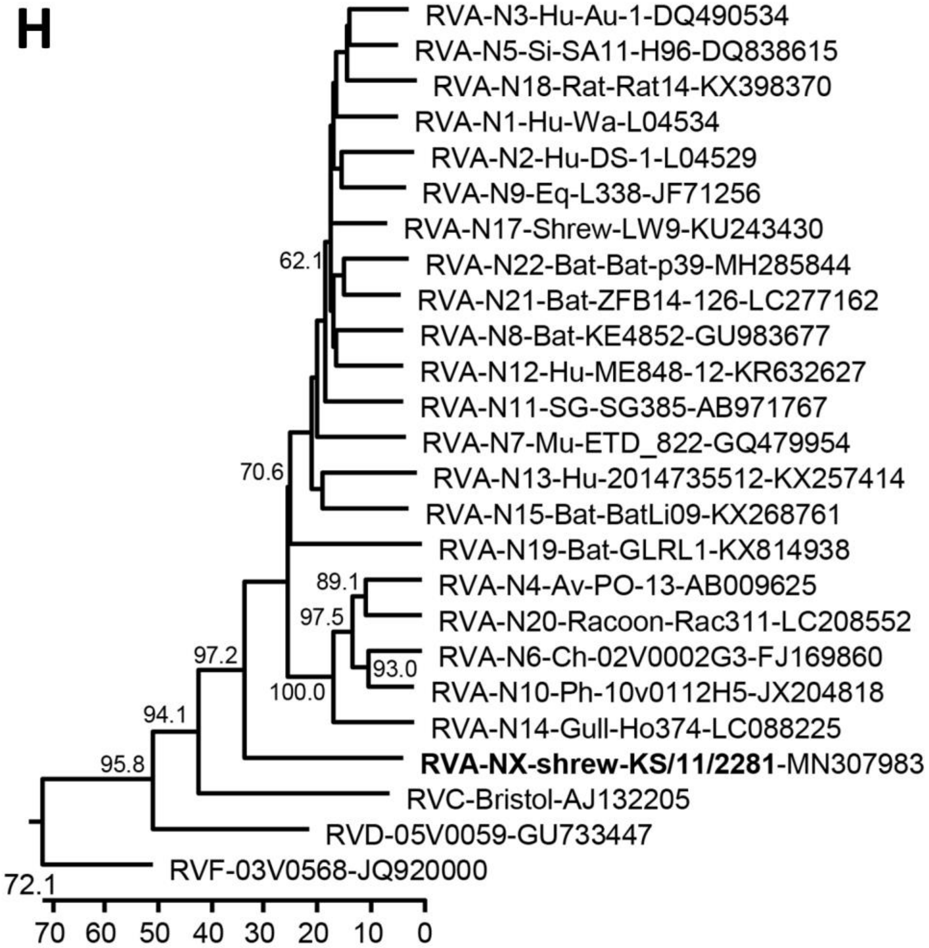
RVA-N3-Hu-Au-1-DQ490534
RVA-N5-Si-SA11-H96-DQ838615
RVA-N18-Rat-Rat14-KX398370
RVA-N1-Hu-Wa-L04534
RVA-N2-Hu-DS-1-L04529
RVA-N9-Eq-L338-JF71256
RVA-N17-Shrew-LW9-KU243430
62.1
RVA-N22-Bat-Bat-p39-MH285844
RVA-N21-Bat-ZFB14-126-LC277162
RVA-N8-Bat-KE4852-GU983677
RVA-N12-Hu-ME848-12-KR632627
RVA-N11-SG-SG385-AB971767
RVA-N7-Mu-ETD_822-GQ479954
70.6
RVA-N13-Hu-2014735512-KX257414
RVA-N15-Bat-BatLi09-KX268761
RVA-N19-Bat-GLRL1-KX814938
89.1
RVA-N4-Av-PO-13-AB009625
97.5
RVA-N20-Racoon-Rac311-LC208552
97.2
RVA-N6-Ch-02V0002G3-FJ169860
93.0
100.0
RVA-N10-Ph-10v0112H5-JX204818
94.1
RVA-N14-Gull-Ho374-LC088225
95.8
RVA-NX-shrew-KS/11/2281-MN307983
RVC-Bristol-AJ132205
RVD-05V0059-GU733447
RVF-03V0568-JQ920000
72.1
70 60 50 40 30 20 10 0

I

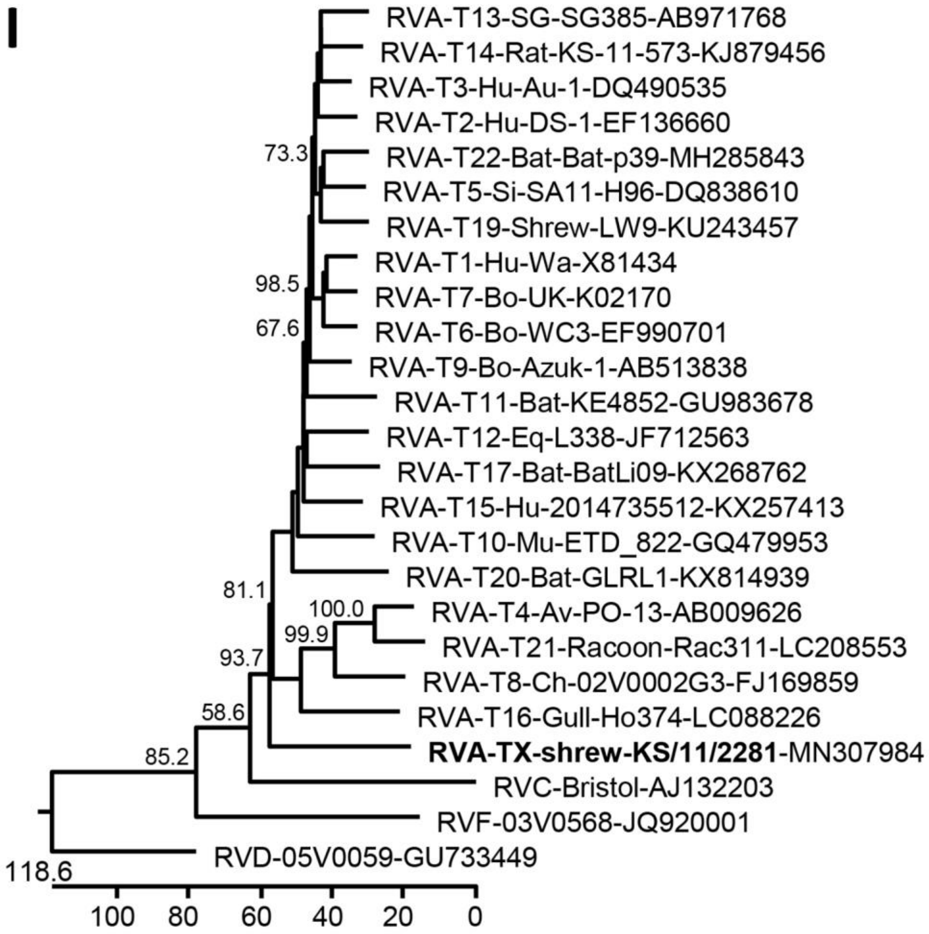
RVA-T13-SG-SG385-AB971768
RVA-T14-Rat-KS-11-573-KJ879456
RVA-T3-Hu-Au-1-DQ490535
RVA-T2-Hu-DS-1-EF136660
73.3
RVA-T22-Bat-Bat-p39-MH285843
RVA-T5-Si-SA11-H96-DQ838610
RVA-T19-Shrew-LW9-KU243457
RVA-T1-Hu-Wa-X81434
98.5
RVA-T7-Bo-UK-K02170
67.6
RVA-T6-Bo-WC3-EF990701
RVA-T9-Bo-Azuk-1-AB513838
RVA-T11-Bat-KE4852-GU983678
RVA-T12-Eq-L338-JF712563
RVA-T17-Bat-BatLi09-KX268762
RVA-T15-Hu-2014735512-KX257413
RVA-T10-Mu-ETD_822-GQ479953
RVA-T20-Bat-GLRL1-KX814939
81.1
100.0
RVA-T4-Av-PO-13-AB009626
99.9
RVA-T21-Racoon-Rac311-LC208553
93.7
RVA-T8-Ch-02V0002G3-FJ169859
58.6
RVA-T16-Gull-Ho374-LC088226
85.2
RVA-TX-shrew-KS/11/2281-MN307984
RVC-Bristol-AJ132203
RVF-03V0568-JQ920001
RVD-05V0059-GU733449
118.6
100
80
60
40
20
0

**J**

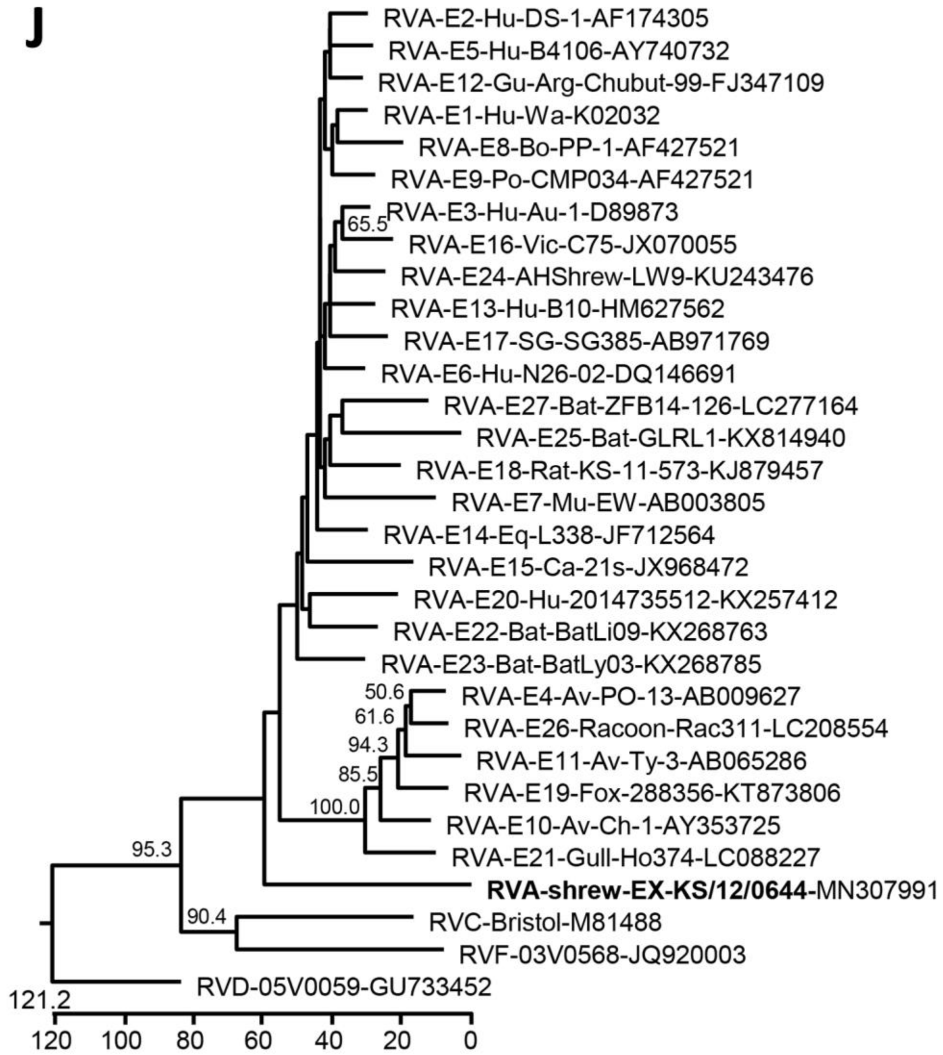

RVA-E2-Hu-DS-1-AF174305
RVA-E5-Hu-B4106-AY740732
RVA-E12-Gu-Arg-Chubut-99-FJ347109
RVA-E1-Hu-Wa-K02032
RVA-E8-Bo-PP-1-AF427521
RVA-E9-Po-CMP034-AF427521
65.5
RVA-E3-Hu-Au-1-D89873
RVA-E16-Vic-C75-JX070055
RVA-E24-AHShrew-LW9-KU243476
RVA-E13-Hu-B10-HM627562
RVA-E17-SG-SG385-AB971769
RVA-E6-Hu-N26-02-DQ146691
RVA-E27-Bat-ZFB14-126-LC277164
RVA-E25-Bat-GLRL1-KX814940
RVA-E18-Rat-KS-11-573-KJ879457
RVA-E7-Mu-EW-AB003805
RVA-E14-Eq-L338-JF712564
RVA-E15-Ca-21s-JX968472
RVA-E20-Hu-2014735512-KX257412
RVA-E22-Bat-BatLi09-KX268763
RVA-E23-Bat-BatLy03-KX268785
50.6
RVA-E4-Av-PO-13-AB009627
61.6
RVA-E26-Racoon-Rac311-LC208554
94.3
RVA-E11-Av-Ty-3-AB065286
85.5
RVA-E19-Fox-288356-KT873806
100.0
RVA-E10-Av-Ch-1-AY353725
RVA-E21-Gull-Ho374-LC088227
95.3
**RVA-shrew-EX-KS/12/0644**-MN307991
90.4
RVC-Bristol-M81488
RVF-03V0568-JQ920003
RVD-05V0059-GU733452
121.2
120 100 80 60 40 20 0

K

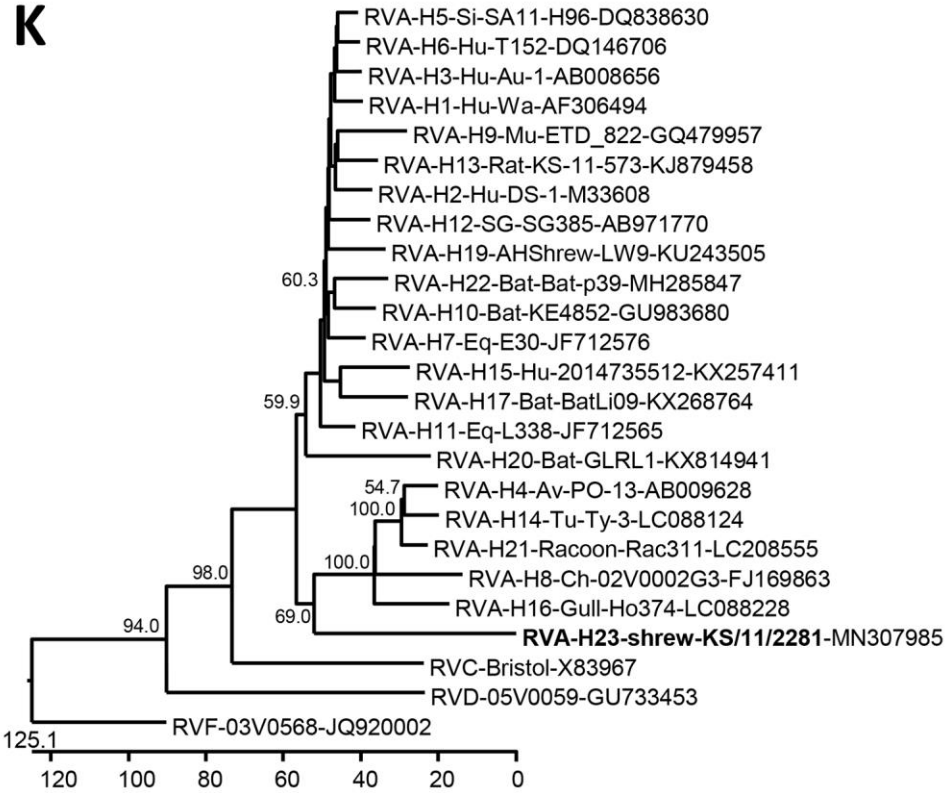


**Appendix Figure 1.** Phylogenetic relationship of the shrew rotavirus A (RVA) with genotype reference strains based on complete or partial nucleotide sequences of the genome segments encoding virus protein (VP) 1 (A), VP2 (B), VP3 (C), VP4 (D), VP6 (E), VP7 (F), nonstructural protein (NSP) 1 (G), NSP2 (H), NSP3 (I), NSP4 (J), and NSP5 (K). The rotavirus genotype, host (for abbreviations see below), strain designation and GenBank accession numbers are indicated at the branches of the tree. Rotavirus C (RVC), D (RVD), and F (RVF) strains are included as outgroups. The shrew virus from this study is given in bold letters. The tree was constructed using a neighbor-joining method implemented in the MEGALIGN module of DNASTAR software package (Lasergene) and a bootstrap analysis with 1000 trials and 111 random seeds. Bootstrap values of >50% are shown. The trees are scaled in nucleotide substitutions per 100 residues. Alp, alpaca; Av, avian; Bo, bovine; Ca, canine; Ch, chicken; Cm, camel; Eq, equine; Gu, guanaco; Hu, human; Mu, murine; Ph, pheasant; Pi, pigeon; Po, porcine; Ra, rabbit; Rh, rhesus monkey; RVA, rotavirus A; RVC, rotavirus C; RVD, rotavirus D; RVF, rotavirus F; SG, sugar glider; AHShrew, Asian house shrew; shrew, common shrew from this study; Si, simian; Tu, turkey; Vic, vicuna.

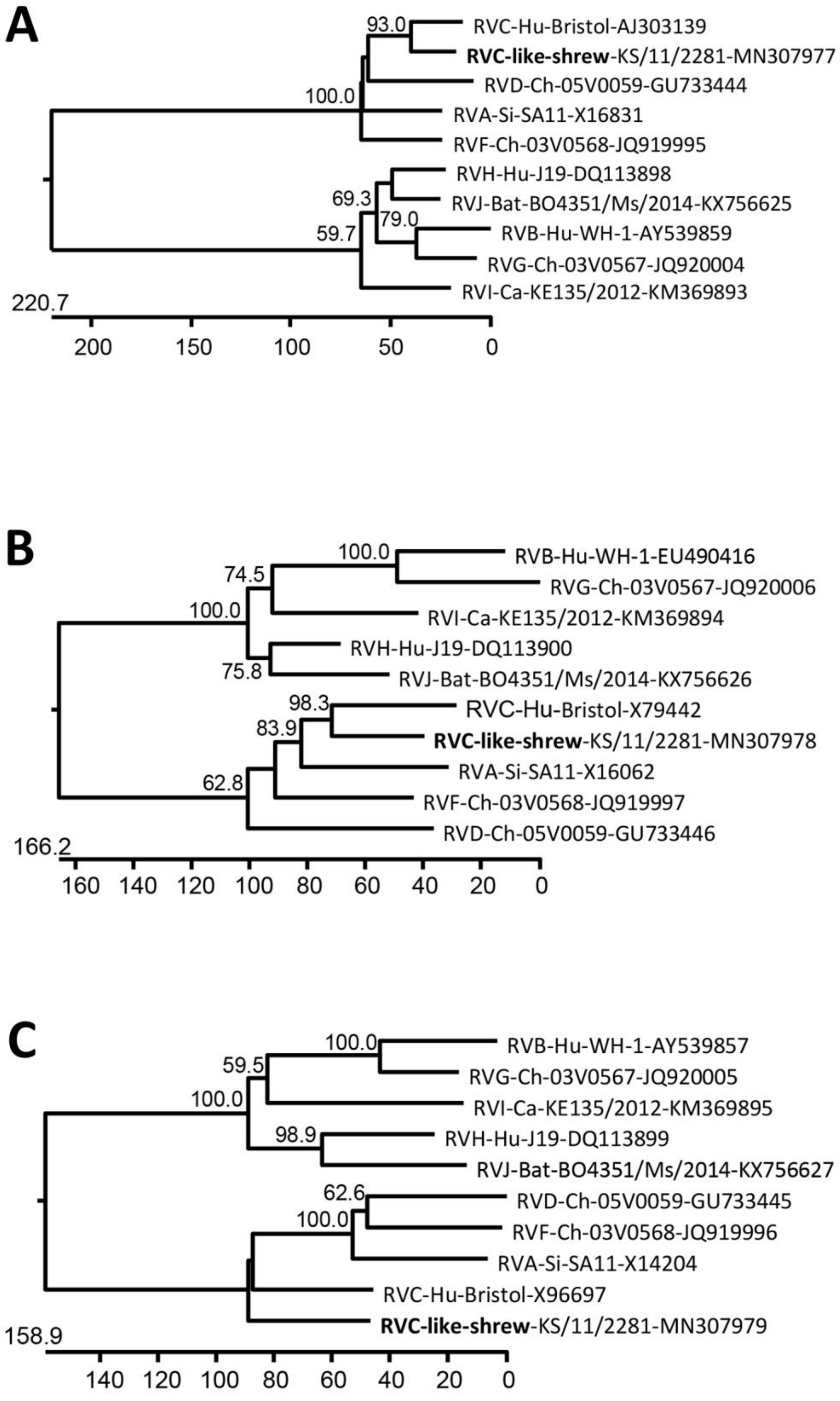
A
93.0
RVC-Hu-Bristol-AJ303139
RVC-like-shrew-KS/11/2281-MN307977
RVD-Ch-05V0059-GU733444
100.0
RVA-Si-SA11-X16831
RVF-Ch-03V0568-JQ919995
RVH-Hu-J19-DQ113898
69.3
RVJ-Bat-BO4351/Ms/2014-KX756625
79.0
59.7
RVB-Hu-WH-1-AY539859
RVG-Ch-03V0567-JQ920004
RVI-Ca-KE135/2012-KM369893
220.7
200 150 100 50 0
B
100.0
RVB-Hu-WH-1-EU490416
74.5
RVG-Ch-03V0567-JQ920006
100.0
RVI-Ca-KE135/2012-KM369894
RVH-Hu-J19-DQ113900
75.8
RVJ-Bat-BO4351/Ms/2014-KX756626
98.3
RVC-Hu-Bristol-X79442
83.9
RVC-like-shrew-KS/11/2281-MN307978
RVA-Si-SA11-X16062
62.8
RVF-Ch-03V0568-JQ919997
RVD-Ch-05V0059-GU733446
166.2
160 140 120 100 80 60 40 20 0
C
100.0
RVB-Hu-WH-1-AY539857
59.5
RVG-Ch-03V0567-JQ920005
100.0
RVI-Ca-KE135/2012-KM369895
98.9
RVH-Hu-J19-DQ113899
RVJ-Bat-BO4351/Ms/2014-KX756627
62.6
RVD-Ch-05V0059-GU733445
100.0
RVF-Ch-03V0568-JQ919996
RVA-Si-SA11-X14204
RVC-Hu-Bristol-X96697
RVC-like-shrew-KS/11/2281-MN307979
158.9
140 120 100 80 60 40 20 0

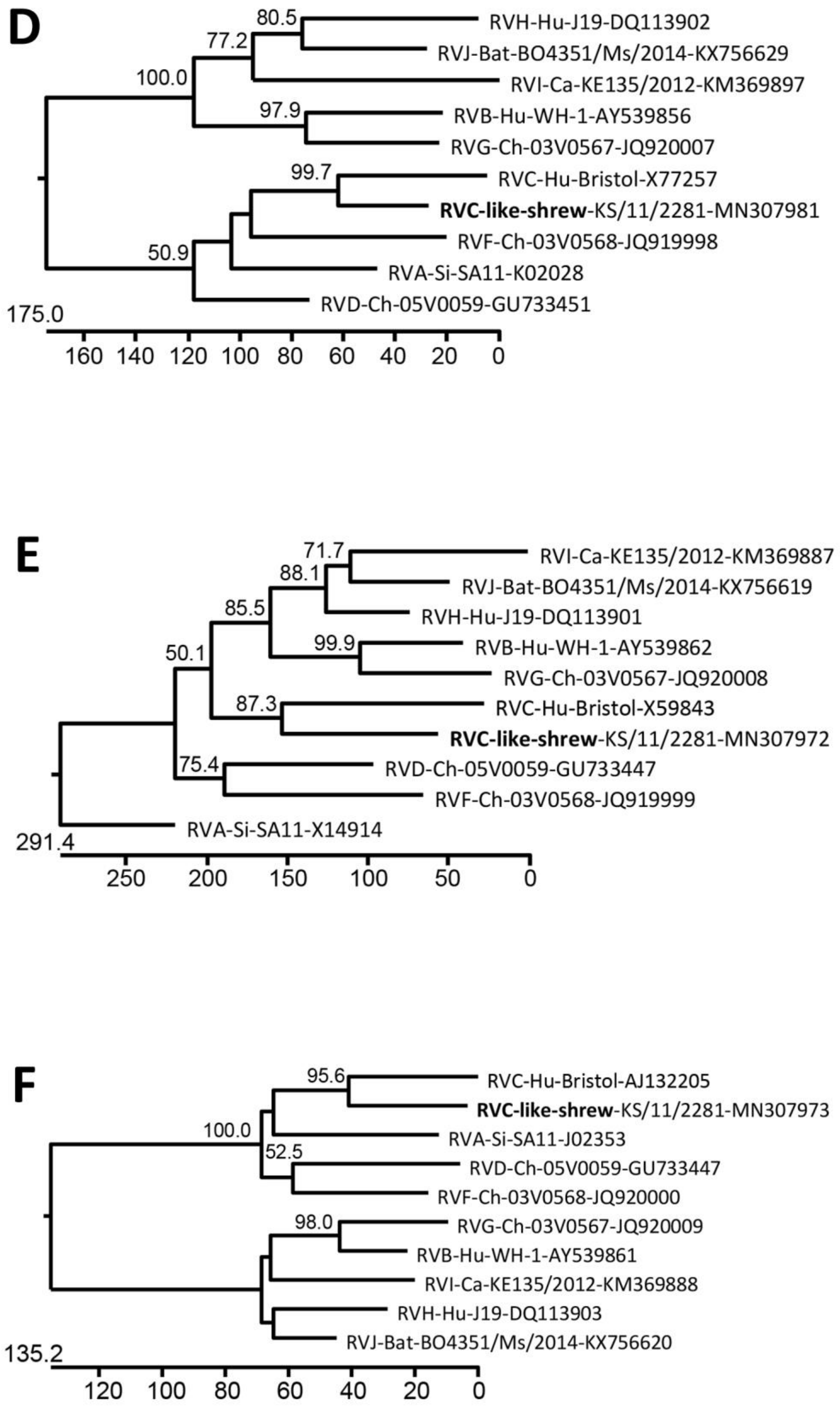
D
RVH-Hu-J19-DQ113902
RVJ-Bat-BO4351/Ms/2014-KX756629
RVI-Ca-KE135/2012-KM369897
RVB-Hu-WH-1-AY539856
RVG-Ch-03V0567-JQ920007
RVC-Hu-Bristol-X77257
RVC-like-shrew-KS/11/2281-MN307981
RVF-Ch-03V0568-JQ919998
RVA-Si-SA11-K02028
RVD-Ch-05V0059-GU733451
80.5
77.2
100.0
97.9
99.7
50.9
175.0
160 140 120 100 80 60 40 20 0
E
RVI-Ca-KE135/2012-KM369887
RVJ-Bat-BO4351/Ms/2014-KX756619
RVH-Hu-J19-DQ113901
RVB-Hu-WH-1-AY539862
RVG-Ch-03V0567-JQ920008
RVC-Hu-Bristol-X59843
RVC-like-shrew-KS/11/2281-MN307972
RVD-Ch-05V0059-GU733447
RVF-Ch-03V0568-JQ919999
RVA-Si-SA11-X14914
71.7
88.1
85.5
99.9
50.1
87.3
75.4
291.4
250 200 150 100 50 0
F
RVC-Hu-Bristol-AJ132205
RVC-like-shrew-KS/11/2281-MN307973
RVA-Si-SA11-J02353
RVD-Ch-05V0059-GU733447
RVF-Ch-03V0568-JQ920000
RVG-Ch-03V0567-JQ920009
RVB-Hu-WH-1-AY539861
RVI-Ca-KE135/2012-KM369888
RVH-Hu-J19-DQ113903
RVJ-Bat-BO4351/Ms/2014-KX756620
95.6
100.0
52.5
98.0
135.2
120 100 80 60 40 20 0

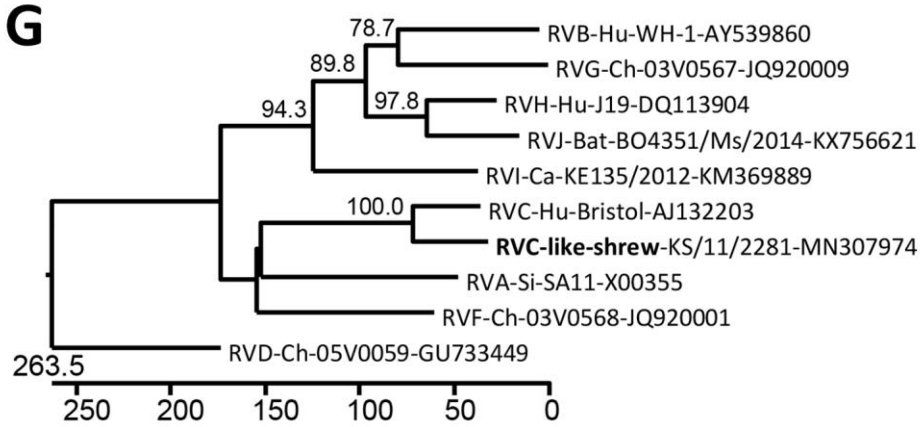


**Appendix Figure 2.** Phylogenetic relationship of the shrew species C-like rotavirus with the rotavirus A-J type species based on the deduced amino acid sequences of partial virus protein (VP) 2 (A), VP3 (B), VP4 (C), VP7 (D), nonstructural protein (NSP) 1 (E), NSP2 (F), and NSP3 (G). The rotavirus species (RVA-RVJ), host (for abbreviations see below), strain designation, and GenBank accession numbers are indicated at the branches of the tree. The shrew virus is given in bold letters. The tree was constructed using a neighbor-joining method implemented in the MEGALIGN module of DNASTAR software package (Lasergene) and a bootstrap analysis with 1000 trials and 111 random seeds. Bootstrap values of >50% are shown. The trees are scaled in amino acid substitutions per 100 residues. aa, amino acid residues; Ca, canine; Ch, chicken; Hu, human; RVA, rotavirus A; RVB, rotavirus B; RVC, rotavirus C; RVD, rotavirus D; RVF, rotavirus F; RVG, rotavirus G; RVH, rotavirus H; RVI, rotavirus I; RVJ, rotavirus J; Si, simian.

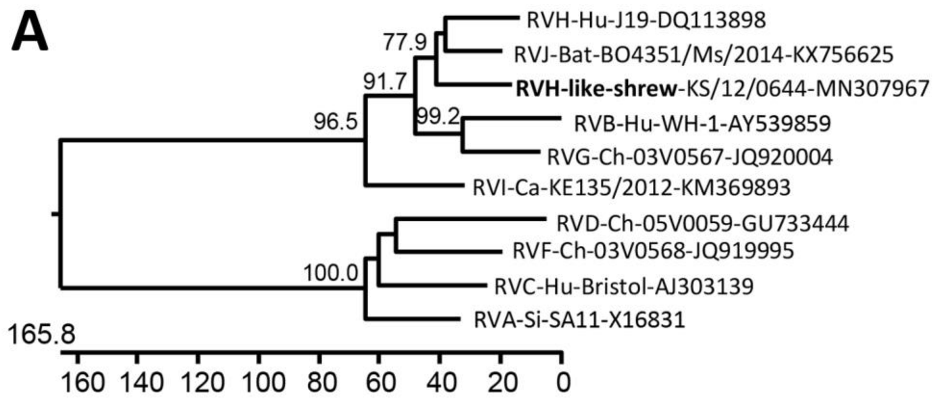

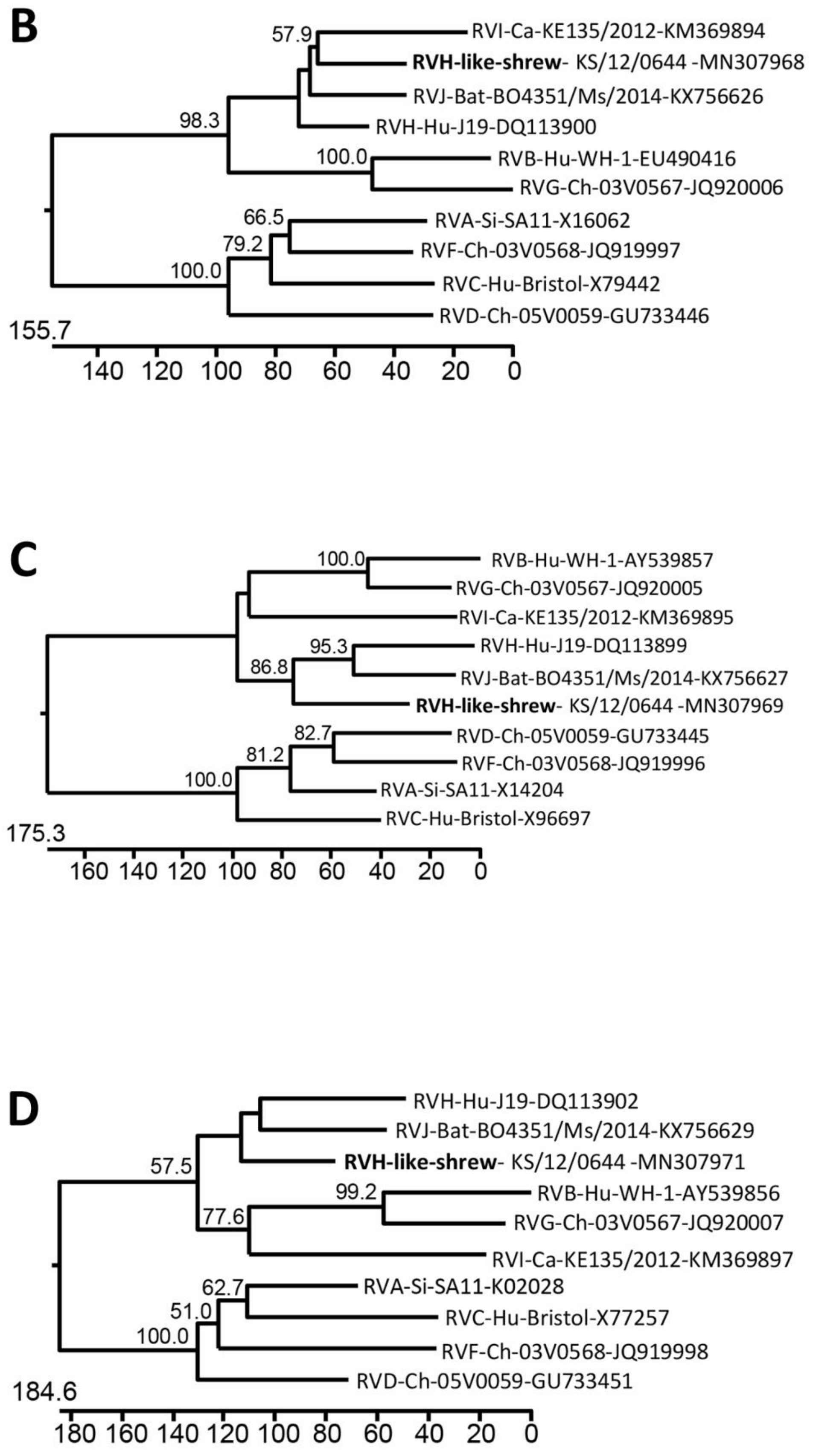
B
57.9
RVI-Ca-KE135/2012-KM369894
RVH-like-shrew- KS/12/0644 -MN307968
RVJ-Bat-BO4351/Ms/2014-KX756626
98.3
RVH-Hu-J19-DQ113900
100.0
RVB-Hu-WH-1-EU490416
RVG-Ch-03V0567-JQ920006
66.5
RVA-Si-SA11-X16062
79.2
RVF-Ch-03V0568-JQ919997
100.0
RVC-Hu-Bristol-X79442
RVD-Ch-05V0059-GU733446
155.7
140 120 100 80 60 40 20 0
C
100.0
RVB-Hu-WH-1-AY539857
RVG-Ch-03V0567-JQ920005
RVI-Ca-KE135/2012-KM369895
95.3
RVH-Hu-J19-DQ113899
86.8
RVJ-Bat-BO4351/Ms/2014-KX756627
RVH-like-shrew- KS/12/0644 -MN307969
82.7
RVD-Ch-05V0059-GU733445
81.2
RVF-Ch-03V0568-JQ919996
100.0
RVA-Si-SA11-X14204
RVC-Hu-Bristol-X96697
175.3
160 140 120 100 80 60 40 20 0
D
RVH-Hu-J19-DQ113902
RVJ-Bat-BO4351/Ms/2014-KX756629
57.5
RVH-like-shrew- KS/12/0644 -MN307971
99.2
RVB-Hu-WH-1-AY539856
77.6
RVG-Ch-03V0567-JQ920007
RVI-Ca-KE135/2012-KM369897
62.7
RVA-Si-SA11-K02028
51.0
RVC-Hu-Bristol-X77257
100.0
RVF-Ch-03V0568-JQ919998
RVD-Ch-05V0059-GU733451
184.6
180 160 140 120 100 80 60 40 20 0

**E**

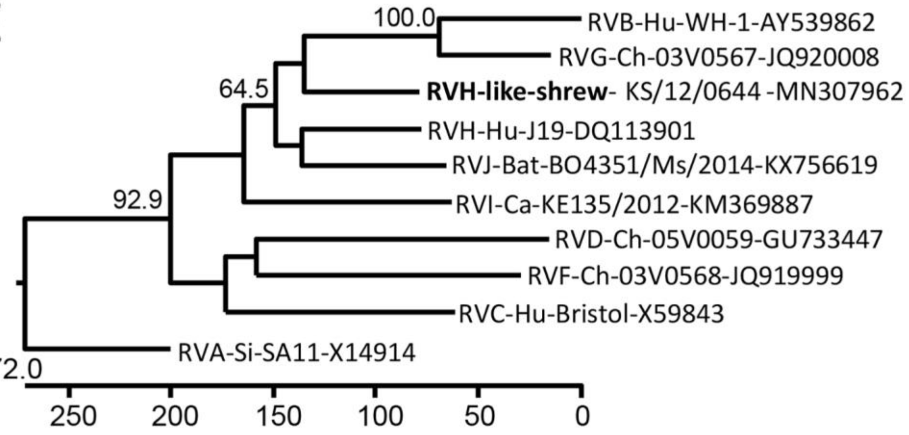
100.0
RVB-Hu-WH-1-AY539862
RVG-Ch-03V0567-JQ920008
64.5
**RVH-like-shrew**- KS/12/0644 -MN307962
RVH-Hu-J19-DQ113901
RVJ-Bat-BO4351/Ms/2014-KX756619
92.9
RVI-Ca-KE135/2012-KM369887
RVD-Ch-05V0059-GU733447
RVF-Ch-03V0568-JQ919999
RVC-Hu-Bristol-X59843
RVA-Si-SA11-X14914
272.0
250
200
150
100
50
0

**F**

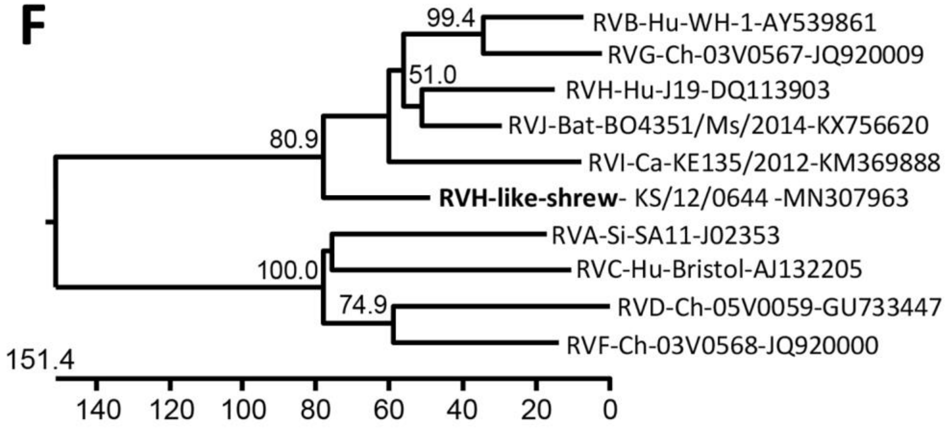
99.4
RVB-Hu-WH-1-AY539861
RVG-Ch-03V0567-JQ920009
51.0
RVH-Hu-J19-DQ113903
RVJ-Bat-BO4351/Ms/2014-KX756620
80.9
RVI-Ca-KE135/2012-KM369888
**RVH-like-shrew**- KS/12/0644 -MN307963
RVA-Si-SA11-J02353
100.0
RVC-Hu-Bristol-AJ132205
74.9
RVD-Ch-05V0059-GU733447
RVF-Ch-03V0568-JQ920000
151.4
140
120
100
80
60
40
20
0

**G**

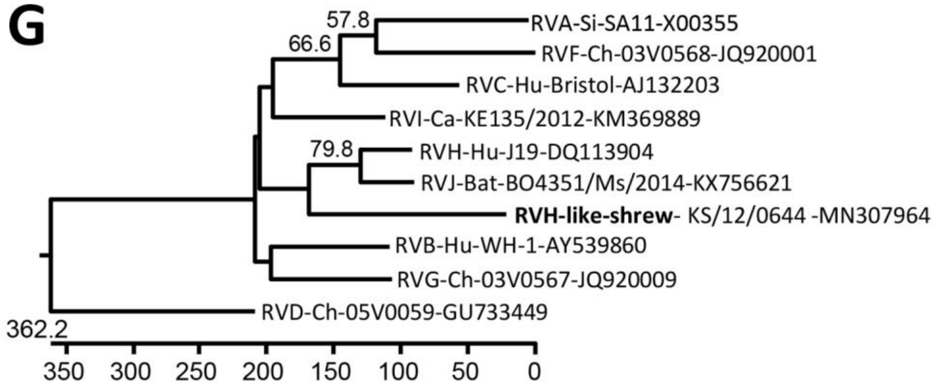
57.8
RVA-Si-SA11-X00355
66.6
RVF-Ch-03V0568-JQ920001
RVC-Hu-Bristol-AJ132203
RVI-Ca-KE135/2012-KM369889
79.8
RVH-Hu-J19-DQ113904
RVJ-Bat-BO4351/Ms/2014-KX756621
**RVH-like-shrew**- KS/12/0644 -MN307964
RVB-Hu-WH-1-AY539860
RVG-Ch-03V0567-JQ920009
RVD-Ch-05V0059-GU733449
362.2
350
300
250
200
150
100
50
0

**Appendix Figure 3.** Phylogenetic relationship of the shrew species H-like rotavirus with the rotavirus A-J type species based on the deduced amino acid sequences of partial or complete virus protein (VP) 2 (A), VP3 (B), VP4 (C), VP7 (D), nonstructural protein (NSP) 1 (E), NSP2 (F), and NSP3 (G). The rotavirus species (RVA-RVJ), host (for abbreviations see below), strain designation, and GenBank accession numbers are indicated at the branches of the tree. The shrew virus is given in bold letters. The tree was constructed using a neighbor-joining method implemented in the MEGALIGN module of DNASTAR software package (Lasergene) and a bootstrap analysis with 1000 trials and 111 random seeds. Bootstrap values of >50% are shown. The trees are scaled in amino acid substitutions per 100 residues. aa, amino acid residues; Ca, canine; Ch, chicken; Hu, human; RVA, rotavirus A; RVB, rotavirus B; RVC, rotavirus C; RVD, rotavirus D; RVF, rotavirus F; RVG, rotavirus G; RVH, rotavirus H; RVI, rotavirus I; RVJ, rotavirus J; Si, simian.

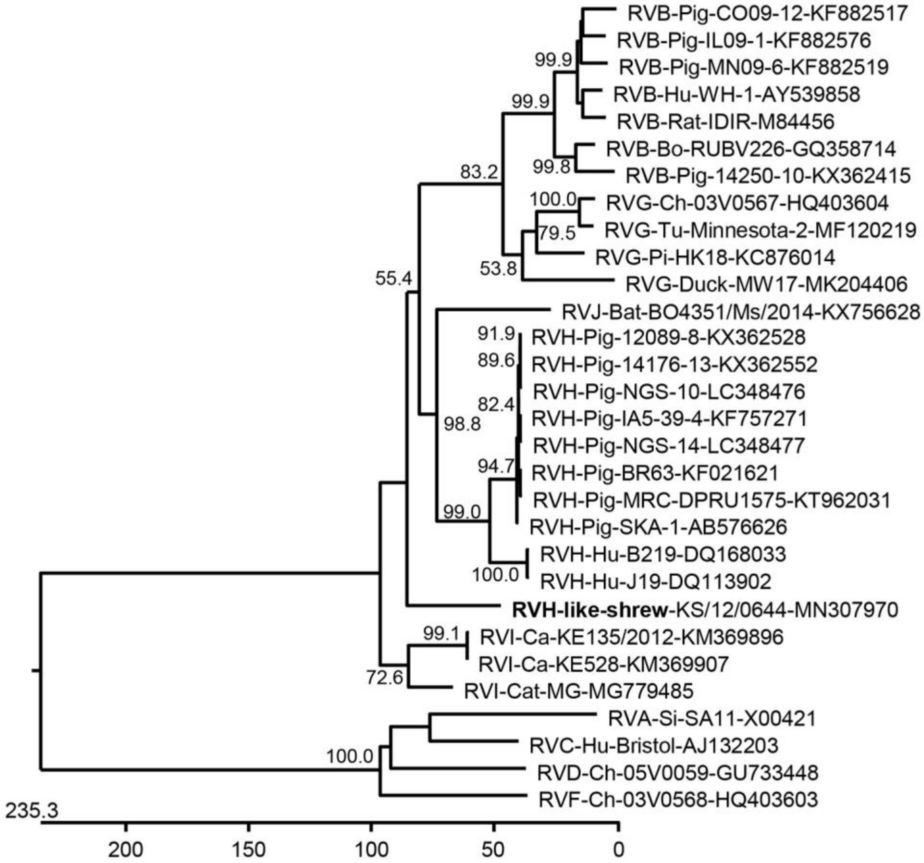


**Appendix Figure 4.** Phylogenetic relationship of the shrew species H-like rotavirus within the rotavirus B/G/H/I/J cluster. Type species of rotavirus A, C, D, and F are included as outgroup. The complete deduced amino acid sequences of VP6 were used for construction of a phylogenetic tree using a neighbor-joining method implemented in the MEGALIGN module of DNASTAR software package (Lasergene) and a bootstrap analysis with 1000 trials and 111 random seeds. Bootstrap values of >50%

are shown. The tree is scaled in amino acid substitutions per 100 residues. The rotavirus species (RVA-RVJ), host (for abbreviations see below), strain designation, and GenBank accession numbers are indicated at the branches of the tree. The shrew virus is given in bold letters. Bo, bovine; Ca, canine; Ch, chicken; Hu, human; Pi, pigeon; RVA, rotavirus A; RVB, rotavirus B; RVC, rotavirus C; RVD, rotavirus D; RVF, rotavirus F; RVG, rotavirus G; RVH, rotavirus H; RVI, rotavirus I; RVJ, rotavirus J; Si, simian; Tu, turkey.
